# Psychometric properties of the Incivility in Nursing Education - Revised Survey - Brazilian version*

**DOI:** 10.1590/1518-8345.6950.4215

**Published:** 2024-08-12

**Authors:** Vanessa dos Santos Ribeiro, Cynthia M. Clark, Claudia Benedita dos Santos, João Marôco, Jonas Bodini Alonso, Aline Helena Appoloni Eduardo, Emilia Campos de Carvalho

**Affiliations:** 1 Universidade de São Paulo, Escola de Enfermagem de Ribeirão Preto, PAHO/WHO Collaborating Centre for Nursing Research Development, Ribeirão Preto, SP, Brazil.; 2 Scholarship holder at the Coordenação de Aperfeiçoamento de Pessoal de Nível Superior (CAPES), Brazil.; 3 Boise State University, School of Nursing, Boise, Idaho, ID, United States of America.; 4 Scholarship holder at the Conselho Nacional de Desenvolvimento Científico e Tecnológico (CNPq), Brazil.; 5 William James Center for Research, ISPA - Instituto Universitário, Lisboa, Portugal.; 6 Universidade Federal de São Carlos, Departamento de Enfermagem, São Carlos, SP, Brazil.

**Keywords:** Nursing, Incivility, Validation Studies, Education, Students, Nursing, Teachers

## Abstract

**Objective:**

to analyze the psychometric properties of the Incivility in Nursing Education - Revised Survey - Brazilian version with undergraduate nursing students.

**Method:**

methodological study conducted in a nursing school in São Paulo state. It is the analysis of the psychometric properties (reliability and construct validity) of the items in the INE-R survey - Brazilian version. Construct validity was performed by Confirmatory Factor Analysis, and reliability by test-retest in order to verify the instrument’s stability, as calculated by the Intraclass Correlation Coefficient and the Internal Consistency of the items according to Cronbach’s alpha, ordinal alpha and McDonalds’s omega coefficients*.*

**Results:**

Confirmatory Factor Analysis fitted the proposed model with two factors (low and high incivility), with a suggestion to exclude one of student items. Most of the fitting values for the student items and all of the faculty-member items complied with the references established in the literature; the values for Internal Consistency Coefficients were greater than 0.80, and Intraclasss Correlation Coefficients were greater than 0.75.

**Conclusion:**

the Brazilian version of the Incivility in Nursing Education - Revised Survey is validated for the studied context, as it has shown satisfactory reliability and validity by means of factor analysis, which has confirmed the original two-factor model, with 23 items addressing student behaviors and 24 items applied to faculty behaviors.

## Highlights

**(1)** Incivility is a global and growing phenomenon in higher education.

**(2)** An uncivil environment interferes with learning and health indicators.

**(3)** A validated survey to measure incivility in nursing education is presented.

## Introduction

For more than four decades, the higher education environment, including that of nursing programs, has been the scenario of uncivil behavior, that is, behavior that does not comply with social rules for social coexistence^1^ *.* Incivility is any behavior that disrupts the learning process and interferes with a cooperative learning environment.

Sometimes described as the use of power over others^2^, incivility can be expressed by behaviors directed at another person, ranging from less aggressive forms, such as insults, disagreements and conflicts, to more serious forms of aggressive behavior, such as physical violence between individuals. Incivility can also represent any conversations, interactions or attitudes that adversely affect the well-being of students or teachers, weakening professional relationships, and thus hindering the teaching-learning process and interfering with the quality of physical and mental health^3^.

If allowed, uncivil behavior can lead to a chain process in which more and more uncivil acts are generated. In this regard, teachers, tutors, and academic managers must be able to reduce such behaviors in different educational environments, preventing incivilities from negatively affecting relationships and learning, both theoretical and practical, thus compromising the training of future nurses^4^ ^)-(^ ^5^.

The presence of incivility, both in the nursing teaching environment and in nursing practice, is a global phenomenon^6^, which has been reported in studies carried out in different cultures, such as Iran^7^ ^)-(^ ^8^, the United Arab Emirates^6^, Afghanistan^9^, Canada^10^, Italy^11^, China^12^, Indonesia^13^, Korea^14^, Turkey^15^ and the United States of America^16^ ^)-(^ ^17^, among others. Although global, a recent multicenter study involving nursing professors from 10 countries found that the phenomenon was perceived differently in the countries participating in the study^18^.

Incivility, or other forms of aggression, occur both in face-to-face relationships and in virtual relationships, which often involve communications in the social media or remote academic activities. In nursing, there has been increased incivility in online courses^19^. This is a complex and multidirectional process in which students, professors and preceptors may contribute to an atmosphere of disrespect for one another or for the learning process, by showing gender-related behaviors^20^, failure to attend classes^21^, unnecessary answers^22^ and impulsive and aggressive acts displayed online^23^, among others. 

Studies have found a positive correlation between stress and incivility^4^ ^),(^ ^23^ ^)-(^ ^24^. During the COVID-19 pandemic, a time when remote classes were intensified, studies reported an increase in stress, anxiety and depression experienced by nursing students^16^.

As highlighted in the conceptual model of incivility in nursing education^25^, based on the perception of the presence of uncivil behaviors, especially by professors, students respond with the possibility of remaining at the institution and conforming as expected (loyalty), challenging the *status quo* and making an effort to change such behaviors at the institution (voice), or leaving the school (exit). Emotional responses, expectations and decisions depend on the support that a student may receive, which will contribute to the successful completion of the program^25^. 

Breaches of the rules agreed upon for the school environment, whether face-to-face or remote, by students or faculty, undermine the teaching-learning process and must be identified, as well as their causes, so that they can be managed and dealt with.

One of the instruments designed to measure uncivil behavior is the Incivility in Nursing Education Survey (INE). When developing INE, and in its successive revisions, the author sought to identify the perceptions of students and professionals concerning the behaviors that would represent the breadth of the incivility phenomenon. Studies carried out in different cultures or with professionals and students from different types of nursing programs show similarities in these behaviors. Evidence used to develop and revise INE-R^3^ describes the basis for including specific student and faculty behaviors. Such behaviors portray a continuum, referred to as the Continuum of Workplace Aggression^26^, which characterizes a reliable structure with a series of uncivil behaviors, ranging from disruptive behaviors or low-level incivility to more serious behaviors, such as physical violence or tragedies. When related to students, those behaviors include distraction or disrespect in the classroom, disrespect for others or a general lack of interest in class^27^. 

INE-R, which was developed in 2004, consists of the definition of incivility, respondents’ identification data, a set of items containing incivility behaviors displayed by faculty members and another set of items containing incivility behaviors expressed by students, as well as evaluative questions. The process of developing INE and those of revising it are described in the literature^28^ ^)-(^ ^29^. In 2014, the original INE-R was revised and renamed as the Incivility in Nursing Education - Revised (INE-R) Survey, in which some items were revised, and two questions added with blank spaces for the discursive recording of respondents’ experiences with academic incivility and the ways to prevent and deal with the problem. The INE-R version was validated among North American nursing students and professors and obtained adequate results in terms of its structure and reliability^3^ ^),(^ ^7^. 

INE-R^3^ has also been validated with Korean^14^ and Arab students^30^ and, given the success of the investigations that have used it and the importance of having a valid and reliable instrument to measure incivility, it was the object of this study, whose objective was to analyze the psychometric properties of the Brazilian version of the Incivility in Nursing Education - Revised Survey with undergraduate nursing students.

## Method

### Study design, site and period

Methodological study concerning the steps for validity verification according to factor structure analysis and reliability, conducted in a public higher nursing education institution in city in the interior of São Paulo state, Brazil, from June 2021 to March 2022. It was preceded by the cultural adaptation and semantic evaluation of INE-R^31^. 

### Participants and selections criteria

All undergraduate nursing students (n=440) regularly enrolled in the institution were invited, except those in their first year, considering that the participants should have previously attended the program for at least 12 months to in order to answer INE-R^3^. Of those eligible, that is, all students in the undergraduate program, except those in the first year, 60% accepted to participate. There were no requests to withdraw during the completion of the instrument.

### Data collection and instruments used

Recruitment and data collection were carried out following consent from the institution and the collegiate body responsible for undergraduate programs. One of the authors invited the students during breaks from academic activities and provided them with the informed consent and data collection instruments. On those occasions, they were informed that they could be randomly selected for the post-test phase, scheduled to occur in approximately 15 days, according to recommendations in the literature^32^.

Afterwards, each student completed the Informed Consent Form and then the INE-R Survey - Brazilian version, individually, in the presence of the researcher. In order to analyze the stability of the instrument, 60 students were randomly selected to complete the survey again. 

The original INE-R Survey^3^ is self-administered and organized into sections, beginning with the conceptualization of incivility and of the academic environment, followed by the respondent’s demographic data (student or faculty member), which are determined according to the criteria of the study being carried out, such as gender, age, ethnic/racial origin, years of training, the program to which the respondent belongs, the faculty member’s position or the student’s term, among others. In this study, the respondent’s age, gender and ethnic-racial identity, and academic term were considered.

The next section presents a list of 48 items divided into two groups, one with 24 items relating to student behavior, and the other with 24 items relating to faculty behavior. Both have two factors - high incivility and low incivility - consisting of nine and fifteen items respectively. Each item is evaluated using a Likert scale corresponding to the level of incivility that the behavior represents to the respondent, with alternatives ranging from 1 to 4, namely: not uncivil; slightly uncivil, moderately uncivil, and highly uncivil. Each item is also evaluated in terms of the frequency of the behavior in the past 12 months, with four alternative answers (scale of 1 to 4), corresponding respectively to: never; rarely; sometimes; and often. Two questions complete the second session, addressing how the respondent perceives the magnitude of the phenomenon in the institution (assessed with four alternatives, ranging from not a problem to a very serious problem), and how each respondent perceives the likelihood of participation by faculty members and students in uncivil acts (assessed with the following alternatives: much greater by faculty members, greater by faculty members, both, greater by students and much greater by students)^3^. 

The last section presents a set of questions and a narrative space, for respondents to describe situations, express their opinions on incivility in nursing education, the uncivil behavior by students and faculty, the level of civility in the institution, the prioritization of strategies to raise the civility level, as well as the causes and consequences of academic incivility^3^.

### Data treatment and analysis

The data for the different variables were entered, in duplicate, into an Excel spreadsheet, forming a structured database. Descriptive statistics were used for the academic term, ethnicity, and gender variables; for the age variable, the mean and median were used as a measure of central tendency, and the standard deviation as a measure of dispersion.

The psychometric properties (reliability and construct validity) of the items in the INE-R Survey - Brazilian version were analyzed. Firstly, data normality was determined by the absolute values of skewness (Sk) and kurtosis (Ku)^33^. Construct validity was assessed by Confirmatory Factor Analysis and reliability by test-retest in order to ascertain the instrument’s stability, as calculated by the Intraclass Correlation Coefficient (ICC) and through the internal consistency of the items, according to Cronbach’s alpha, ordinal and McDonald’s omega coefficients^32^. The ratio of the number of participants to the number of items in the instrument limited further analysis.

Confirmatory Factor Analysis was carried out using the R software (R Core Team, 2021), version 3.4.2, and the Latent Variable Analysis (LAVAAN) package^34^. The Diagonally Weighted Least Squares (DWLS) method was used to estimate the parameters^35^.

With regard to analyzing the quality of the model’s fit, absolute fit indices, incremental fit indices and population discrepancy indices are taken as alternatives for testing the fit^35^. The population discrepancy index investigated was the Root Mean Square Error of Approximation (RMSEA), which estimates whether the model parameters reproduce the population covariance and, under such conditions, RMSEA tends to be equal to zero. The Standardized Root Mean Square Residual (SRMR) was also evaluated, i.e. the standardized difference between the observed and predicted correlation. Values below 0.10 indicate an acceptable adjustment for RMSEA, and those below 0.08 are considered to be acceptable for SRMR^35^. 

Regarding incremental fit indices, the Normed Fit Index (NFI), the Comparative Fit Index (CFI) and the Tucker-Lewis Index (TLI) were used. The NFI and CFI values, as well as TLI, can be between zero and one, and values above 0.90 indicate an acceptable fit^35^.

Internal consistency as determined by the Cronbach’s alpha coefficient (α) was used to analyze reliability. Such an indicator will make it possible to compare the results obtained from other studies that have validated the instrument. In addition, the values of the ordinal alpha and McDonald’s omega (ω) coefficients were also analyzed for each of the instrument’s domains (high and low level of incivility) in each group of items (those relating to student and those relating to faculty behaviors). The values of Cronbach’s α, ordinal alpha and McDonald’s omega vary between zero and one and, in this study, values equal to or greater than 0.70 were considered acceptable^32^. 

In order to ascertain the stability of the measure, test-retest was carried out, and the Intraclass Correlation Coefficient (ICC) was obtained, as in other studies that have validated this instrument. The significance level for this analysis was 5% (α = 0.05). ICC values between 0.70 and 0.90 are considered to be good and those greater than 0.90 are considered to be excellent^32^.

### Ethical aspects

Participants were invited to take part and assured total privacy of their information. The study was only initiated after approval by the Teaching Committee and the Ethics Committee for Research Involving Human Beings (CEP), both of which were part of the institution where the study was carried out (CEP Report: 3.635.814 and Certificate of Submission for Ethical Appraisal-CAAE 18508919.4.0000.5393).

## Results

A total of 264 students participated in the study. With regard to gender and ethnic-racial identity, 222 (84.1%) identified themselves as women; 40 (15.2%) as men, one (0.4%) as both and another (0.4%) as neither; 180 (68.2%) self-reported as being White, 57 (21.6%) as *Pardo*, 22 (8.3%) as Black, 4 (1.5%) as Asian, while 1 (0.4%) self-identified as Indigenous. Of the students, 177 (67.0%) were from the intermediate years (third to sixth terms) and 87 (33.0%) were from the final years (seventh term onwards).

The mean values, respective standard deviations, skewness (Sk) and kurtosis (Ku) of the behavioral incivility levels when displayed by students and faculty, according to the participants (n = 264), are shown in Table 1. When considering the skewness and kurtosis values for each item, they are lower than three and seven, respectively, for all the items, thus showing that normality was not violated, which is a prerequisite for carrying out CFA.

**Table 1:** Descriptive statistics (means, standard deviation, skewness, and kurtosis) of behavioral incivility levels when displayed by students and faculty, according to participants (n = 264). Interior of São Paulo state, Brazil, 2021-2022

Behaviors	Item	Mean	Standard deviation	Skewnes*s*	Kurtosis
**Related to students**	01	2.76	0.84	-0.33	-0.45
	02	3.17	1.11	-0.94	-0.65
	03	2.87	0.82	-0.37	-0.37
	04	2.21	0.93	0.40	-0.70
	05	3.12	0.82	-0.77	0.15
	06	2.64	0.86	-0.27	-0.57
	07	2.48	0.84	-0.08	-0.60
	08	2.58	0.85	-0.01	-0.65
	09	2.49	0.92	0.10	-0.85
	10	3.08	1.01	-0.73	-0.70
	11	3.02	1.08	-0.72	-0.85
	12	3.17	0.85	-0.81	-0.01
	13	3.31	0.93	-1.13	0.12
	14	3.43	1.10	-1.56	0.61
	15	2.21	1.00	0.40	-0.90
	16	3.14	1.10	-0.94	-0.57
	17	3.05	1.12	-0.77	-0.88
	18	2.54	0.95	-0.10	-0.93
	19	3.42	1.11	-1.56	0.63
	20	3.51	1.08	-1.80	1.33
	21	3.44	1.04	-1.58	0.87
	22	3.47	1.12	-1.69	0.91
	23	3.45	1.13	-1.63	0.75
	24	3.47	1.14	-1.69	0.88
**Related to faculty members**	01	3.28	0.86	-1.08	0.04
	02	3.49	0.95	-1.05	-0.08
	03	3.31	0.86	-1.48	1.42
	04	3.09	0.92	-1.84	1.91
	05	3.01	0.96	-0.73	-0.33
	06	2.87	0.85	-2.23	4.02
	07	2.60	0.94	-2.40	4.48
	08	3.26	0.96	-1.56	1.87
	09	3.22	0.99	-1.49	0.97
	10	3.49	0.78	-2.74	6.71
	11	3.53	0.95	-0.91	0.16
	12	3.01	0.94	-1.82	1.49
	13	3.64	0.78	-2.01	2.21
	14	3.66	0.82	-1.87	1.73
	15	3.46	0.81	-1.88	1.58
	16	3.40	0.96	-1.86	1.56
	17	3.73	0.71	-1.88	1.60
	18	3.21	0.85	-1.08	0.04
	19	3.51	1.05	-1.05	-0.08
	20	3.57	1.01	-1.48	1.42
	21	3.53	1.02	-1.84	1.91
	22	3.53	1.08	-0.73	-0.33
	23	3.52	1.07	-2.23	4.02
	24	3.53	1.07	-2.40	4.48

When considering the domains, the mean levels of incivility for student behaviors were: Low Incivility Domain x = 2.76; SD = 0.53; High Incivility Domain x = 3.39, SD = 0.97; and for faculty members, they were: Low Incivility Domain x = 3.22; SD = 0.69; High Incivility Domain x = 3.58; SD = 0.89. The median of the incivility items attributed to students for the Low Incivility Domain was 3, and for the High Incivility Domain, it was 4; and that attributed to faculty members’ behaviors for the Low Incivility Domain was 3, and for the High Incivility Domain, it was 4.

With regard to the frequency of uncivil behaviors in the past 12 months, the means and respective standard deviations for the items relating to faculty behaviors were: Low Incivility Domain x = 2.04; SD = 0.49; High Incivility Domain x = 1.46, SD = 0.35; and for those relating to students were Low Incivility Domain x = 2.43, SD = 0.53; High Incivility x = 1.54, SD = 0.49. The medians, minimum and maximum values for all the items were the same for students and faculty (median = 2; minimum = 1; maximum = 4), low and high incivility.

In CFA, among the student behaviors, all the items in the High Incivility domain showed high factor loadings, ranging from 0.734 to 0.999. For the Low Incivility Domain, three items (3, 4 and 8) had loadings ranging from 0.304 to 0.396, and the others showed values ranging from 0.613 to 0.825, except for items 1, 5 and 15 which had factor loadings < 0.300. Item 1- expressing disinterest, boredom or apathy regarding course content or subject matter - showed a factor loading of 0.149, CI[95%]: [0.105; 0.192]; item 5- using a computer, cell phone or other electronic devices during a class, meeting or activity for unrelated purposes - showed a factor loading of 0.290, CI[95%]: [0.249; 0.332]; and item 15- demanding make-up tests, deadline extensions or other special favors - showed a factor loading of 0.299; CI[95%]: [0.259; 0.338] (Table 2).

**Table 2:** Results of the Confirmatory Factor Analysis in relation to the factor loadings of the items referring to student behaviors (n = 264). Interior of São Paulo state, Brazil, 2021-2022

Domain	Item	Factor loading	95% Confidence interval	Standard-error	Z*	***P-*value**
**High Incivility**	Item13	0.734	(0.702; 0.766)	0.039	18.916	0.000
	Item 14	0.946	(0.930; 0.962)	0.016	57.785	0.000
	Item17	0.855	(0.833; 0.877)	0.023	37.411	0.000
	Item 19	0.988	(0.983; 0.993)	0.004	236.035	0.000
	Item 20	0.999	(0.997; 1.000)	0.001	1431.998	0.000
	Item 21	0.958	(0.947; 0.969)	0.011	85.421	0.000
	Item 22	0.999	(0.997; 1.000)	0.001	941.880	0.000
	Item 23	0,999	(0,997; 1.000)	0,001	1210,095	0,000
	Item 24	0.999	(0.997; 1.000)	0.001	740.119	0.000
**Low Incivility**	Item 1	0.149	(0.105; 0.192)	0.070	2.119	0.000
	Item 2	0.802	(0.772; 0.831)	0.032	24.995	0.000
	Item 3	0.350	(0.311; 0.388)	0.060	5.861	0.000
	Item 4	0.304	(0.261; 0.346)	0.066	4.618	0.000
	Item 5	0.290	(0.249; 0.332)	0.062	4.653	0.000
	Item 6	0.641	(0.604; 0.678)	0.043	15.021	0.000
	Item 7	0.538	(0.500; 0.576)	0.048	11.188	0.000
	Item 8	0.396	(0.354; 0.438)	0.060	6.643	0.000
	Item 9	0.577	(0.537; 0.616)	0.050	11.587	0.000
	Item 10	0.825	(0.798; 0.853)	0.028	29.948	0.000
	Item 11	0.825	(0.798; 0.852)	0.030	27.392	0.000
	Item 12	0.758	(0.728; 0.788)	0.034	22.184	0.000
	Item 15	0.299	(0.259; 0.338)	0.062	4.801	0.000
	Item 16	0.776	(0.746; 0.805)	0.036	21.609	0.000
	Item 18	0.613	(0.581; 0.644)	0.041	14.809	0.000

*Z = Standardized normal variable

As for faculty behaviors, the lowest factor loading of the items was 0.596 (item 18), which belongs to the Low Incivility Domain, and the highest (items 20, 22, 23 and 24) belong to the High Incivility Domain; therefore, all items had satisfactory factor loadings (Table 3).

**Table 3:** Results of the Confirmatory Factor Analysis in relation to the factor loadings of the items relating to faculty behaviors (n = 264). Interior of São Paulo state, Brazil, 2021-2022

Domain	Item	Factor Loading	95% Confidence interval	Standard error	Z*	***P-*value**
**High Incivility**	Item13	0.888	(0.870; 0.906)	0.026	33.940	0.000
	Item 14	0.963	(0.952; 0.975)	0.013	73.082	0.000
	Item17	0.948	(0.935; 0.961)	0.016	59.428	0.000
	Item 19	0.990	(0.985; 0.995)	0.004	241.701	0.000
	Item 20	0.997	(0.995; 0.999)	0.002	606.938	0.000
	Item 21	0.993	(0.989; 0.996)	0.003	313.800	0.000
	Item 22	1.000	(1.000; 1.000)	0.001	1203.589	0.000
	Item 23	0.998	(0.995; 1.000)	0.001	729.320	0.000
	Item 24	0.998	(0.996; 1.000)	0.001	739.529	0.000
**Low Incivility**	Item 1	0.827	(0.807; 0.848)	0.026	31.722	0.000
	Item 2	0.930	(0.914; 0.946)	0.020	47.166	0.000
	Item 3	0.674	(0.647; 0.701)	0.043	15.696	0.000
	Item 4	0.816	(0.706; 0.836)	0.029	28.355	0.000
	Item 5	0.807	(0.786; 0.827)	0.024	33.571	0.000
	Item 6	0.762	(0.739; 0.785)	0.028	26.902	0.000
	Item 7	0.709	(0.684; 0.734)	0.036	19.748	0.000
	Item 8	0.899	(0.882; 0.917)	0.018	51.310	0.000
	Item 9	0.891	(0.873; 0.909)	0.020	44.766	0.000
	Item 10	0.779	(0.756; 0.803)	0.033	23.809	0.000
	Item 11	0.937	(0.921; 0.954)	0.017	54.505	0.000
	Item 12	0.814	(0.794; 0.834)	0.026	31.101	0.000
	Item 15	0.782	(0.758; 0.805)	0.034	23.185	0.000
	Item 16	0.900	(0.883; 0.917)	0.020	45.231	0.000
	Item 18	0.596	(0.567; 0.625)	0.050	12.002	0.000

*Z = Standardized normal variable

Based on the point and interval estimates of the factor loadings of the items in the Brazilian version of the instrument, we suggest a structural organization of the items with two factors, high incivility and low incivility, similarly to the original structure, with the exclusion of item 1 for students, which showed a 95%CI for the factor loading including only values lower than 0.30.

The results of the Confirmatory Factor Analysis in relation to the factor loadings of the items relating to student behaviors, with the exclusion of item 1 for students, are shown in Table 4.

**Table 4:** Results of the Confirmatory Factor Analysis in relation to the factor loadings of the items relating to student behaviors (n = 264). Interior of São Paulo state, Brazil, 2021-2022

Domain	Item	Factor loading	95% Confidence interval	Standard error	Z*	***P-*value**
**High Incivility**	Item13	0.734	(0.702; 0.766)	0.039	18.865	0.000
	Item 14	0.946	(0.930; 0.962)	0.016	57.844	0.000
	Item17	0.855	(0.834; 0.877)	0.023	37.411	0.000
	Item 19	0.988	(0.983; 0.993)	0.004	236.087	0.000
	Item 20	0.999	(0.997; 1.000)	0.001	1431.743	0.000
	Item 21	0.958	(0.947; 0.969)	0.011	85.376	0.000
	Item 22	0.999	(0.997; 1.000)	0.001	941.885	0.000
	Item 23	0.999	(0.997; 1.000)	0.001	1210.134	0.000
	Item 24	0.999	(0.997; 1.000)	0.001	740.191	0.000
**Low Incivility**	Item 2	0.801	(0.771; 0.830)	0.032	24.968	0.000
	Item 3	0.346	(0.307; 0.384)	0.060	5.768	0.000
	Item 4	0.301	(0.258; 0.343)	0.066	4.565	0.000
	Item 5	0.287	(0.246; 0.328)	0.063	4.579	0.000
	Item 6	0.640	(0.604; 0.677)	0.043	14.967	0.000
	Item 7	0.535	(0.497; 0.572)	0.048	11.058	0.000
	Item 8	0.394	(0.352; 0.436)	0.060	6.579	0.000
	Item 9	0.575	(0.536; 0.615)	0.050	11.535	0.000
	Item 10	0.825	(0.798; 0.852)	0.028	29.942	0.000
	Item 11	0.824	(0.797; 0.852)	0.030	27.339	0.000
	Item 12	0.757	(0.728; 0.787)	0.034	22.143	0.000
	Item 15	0.299	(0.259; 0.338)	0.062	4.794	0.000
	Item 16	0.775	(0.746; 0.805)	0.036	21.579	0.000
	Item 18	0.612	(0.580; 0.644)	0.041	14.791	0.000

*Z = Standardized normal variable

In view of the results shown in Table 4, with the exclusion of item 1, and for the reasons given above, we chose to maintain all the other 23 items for students.

For construct evaluation, based on Confirmatory Factor Analysis, the measures adopted to fit the model to the population, tested for the items that portray student and faculty behaviors, respectively, were: the Chi square (χ2 = 815.609 and 491.440); Degrees of Freedom (DF= 229; 251); (χ^2^/DF = 3.56 and 1.96); *p*-values (< 0.001; < 0.001); the Comparative Fit Index (CFI = 1.000; 1.000); the Tucker-Lewis Index or Non-Normalized Fit Index (TLI = 1.000; 1.000); the Normalized Fit Index (NFI = 1.000; 1.000); the Root Mean Square Error of Approximation (RMSEA = 0.092; 0.044); a 95% Confidence Interval for RMSEA (0.083; 0.101) and (0.035; 0.055) and the Standardized Root Mean Square Residuals (SRMR = 0.107; 0.053). Most of the indices for students and all the indices for faculty indicate an acceptable fit.

**Figure 1:**
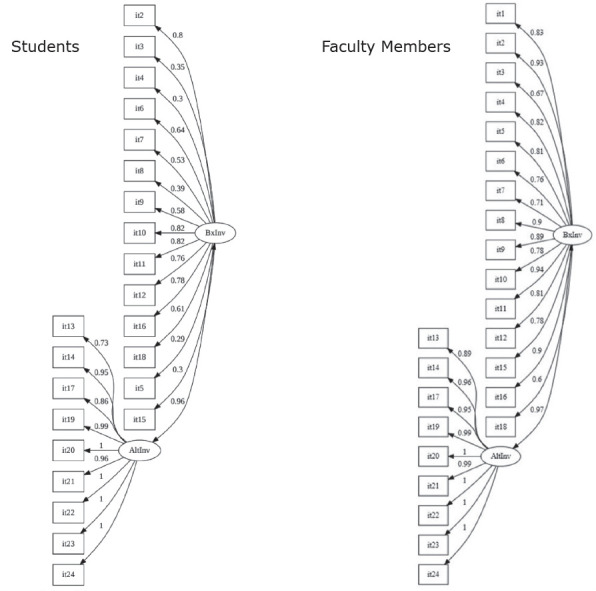
Path diagrams of the Low Incivility and High Incivility Domains of the Incivility in Nursing Education - Revised Survey - Brazilian version, considering the original factor structure. Interior of São Paulo state, Brazil, 2021-2022 *It = Items; ^†^BxInv = Low incivility level; ^‡^AltInv = High incivility level

The representations of the Low Incivility and High Incivility Domains and their items from INE-R - Brazilian version, with their respective factor loadings, from the Confirmatory Factor Analysis, are shown in Figure 1. The structure is presented for faculty members, as proposed in the original model of the instrument, and for students, respecting the justifications contained in the discussion of the items.

The reliability of the INE-R Survey - Brazilian version was evaluated by internal consistency and reproducibility. The internal consistency, assessed by Cronbach’s alpha, ordinal alpha, and McDonald’s omega coefficients, is shown in Table 5.

**Table 5:** Internal consistency of the Incivility in Nursing Education - Revised Survey - Brazilian version, according to the group of incivility behaviors, the dimensions of incivility and the items that comprise them (n = 264). Interior of São Paulo state, Brazil, 2021-2022

Group of behaviors	Incivility dimensions	Items	Cronbach’s alpha value (95% CI)	Alfa ordinal value	Omega value
**Students**	High	13, 14, 17, 19, 20, 21, 22, 23, 24	0.966 (0.962; 0.974)	0.984	0.976
	Low	2 to 12, 15, 16, 18	0.853 (0.827; 0.879)	0.880	0.857
**Faculty**	High	13, 14, 17, 19, 20, 21, 22, 23, 24	0.977 (0.973; 0.981)	0.993	0.989
	Low	1 to 12, 15, 16, 18	0.949 (0.940; 0.958)	0.964	0.951

Internal consistency can be considered highly satisfactory for the items assessing student and faculty behaviors, for both incivility domains, showing adequate reliability in the sample studied.

As for the analysis of the instrument’s reproducibility, of the 60 students selected for this phase, only 29 participated. Regarding the level of incivility, stability, as analyzed by the Intraclass Correlation Coefficient, was considered to be in good agreement, with an ICC value in relation to the items for student behaviors (ICC = 0.612, CI [95%]: [0.330; 0.796] and p-value < 0.0001), and moderate for faculty behaviors (ICC = 0.598 CI[95%]:[0.301; 0.789] and *p*-value < 0.0001).

For the stability of the items portraying the frequency of uncivil behaviors observed in the past 12 months, in the pre- and post-test phases, agreement was considered to be moderate for students (ICC 0.474, CI[95%]: [0.148; 0.710] and *p*-value = 0.003), and good for faculty (ICC = 0.615, CI[95%]: [0.332; 0.789] and p-value < 0.0001). 

## Discussion

This study analyzed the psychometric properties of the Brazilian version of the INE-R Survey^3^. The INE-R survey has been useful to identify the perceptions of uncivil acts by nursing students and faculty members^18^, thus making it possible to distinguish between the perceptions of the two groups^7^. 

As regards the instrument, the study variable is a latent variable, measured by answer choices on a Likert-type scale ranging from 1 to 4. It is, therefore, not symmetrical (there is no neutral point equidistant from the extremes) and not homogeneous in terms of the number of items, as each domain contains, respectively, nine and 15 items^36^. 

Data collection occurred during the COVID-19 pandemic, when students’ and faculty members’ activities were undergoing adjustments, with the introduction of online courses. Such a situation portrayed an overload of demands and difficulties in accessing and handling devices^37^. According to the literature, changes in perceptions of the frequency or level of incivility in student and faculty behavior were identified during the pandemic period^38^ ^)-(^ ^39^.

In this study, the items corresponding to student and faculty behaviors were evaluated only by students. The original North American study validated the instrument by faculty members and students and for the sets of items for student and faculty behaviors^3^. The others validated the entire instrument only with students^14^ or only the items relating to student behaviors^30^. The instrument can be evaluated for each of the focus subjects (students or faculty) or jointly^3^.

The perceptions regarding faculty behavior reported in this study relate to experiences with professors at a public, secular (non-religious) institution, most of whom have worked at the same institution for long periods. The literature cites a study conducted in South Africa^40^, in which working at the same institution was associated with an increase in incivility, and another study which mentions increased levels of incivility in academia^41^. These aspects may have had an impact on the expression of uncivil behavior.

Therefore, the prolonged nature of the professors’ relationship with the institution^40^, the fact that data collection occurred during a period of adjustment to new demands imposed by the pandemic^16^, and the increased use of remote technologies for classes^38^ ^,^ ^42^ during or in the semesters prior to the study may have had an impact on the students’ answers, i.e. their perception of the frequency or level of uncivil behavior.

With regard to the number of participants, when using an instrument such as the one previously described, it is recommended that from 5 to 10 respondents per question should be considered when carrying out factor analysis. However, when using a structural equation model, the literature recommends from 10 to 15 participants per variable in the model^36^. In this study, the sample size of 264 met the first condition, but made it impossible to analyze the model’s factor invariance. In the original study, the sample consisted of 310 North American students^3^; the study that validated the instrument for the Korean culture had 284 participants^14^ and the Arab sample consisted of 389 respondents^30^. All of such studies used factor analysis. 

Concerning the descriptive analysis of this instrument, we chose to present the participants’ perception of the level of incivility and its frequency using the median, even though we had described the mean for the purpose of comparison with studies that have validated or used that instrument for other cultures^14^ ^),(^ ^30^. This indicator (mean) may not clearly represent the variation in the sample because it is a Likert-type scale, hence an ordinal qualitative scale, according to the measurement level^36^.

As a comparison of the incivility levels, for student behaviors, this study showed an overall mean of 3.0, while in the Korean and Arab studies, the means were x = 3.11 and x = 3.45, respectively^14^ ^),(^ ^30^; and for faculty behaviors, our results were x = 3.35, while the Korean study showed x = 3.17(14). The mean frequencies of behavior occurrences in the past 12 months, as reported in our study for student and faculty behaviors, were 2.10 and 1.82, respectively, while in the Arab study, the overall mean for student items was 1.99^30^.

Furthermore, the descriptive analysis of the data in this study revealed some aspects that should be considered. One of them concerns the distribution of answers. The ceiling/floor effect was identified for several items in the INE-R Survey - Brazilian version. This suggests that the distribution of scores is asymmetrical, i.e. it reflects the percentage of participants who scored at the lowest or highest levels of the measure^32^. In a study using INE, the ceiling-floor effect was also observed, with more than 15% frequency for the perception of student and faculty behaviors at both ends of the scale used^29^.

It is acknowledged that disruptive behaviors are more prevalent than high incivility^29^. And rude non-verbal behavior and humiliating comments are more likely to occur than threatening behavior or violent acts^3^.

The tendency identified for participants to attribute a higher level of incivility to the same behavior when it is engaged in by faculty members than when it is shown by students had also been described in a study that analyzed the moderating effect of social hierarchy on uncivil behavior at work^43^, showing that perceived incivility was higher among uncivil acts perpetrated by managers as compared to perceived incivility if perpetrated by a peer.

As for validity and reliability, the literature recommends different alternatives for analyzing psychometric properties, depending on the nature of the variable, form of measurement, sample size and characteristics of the data obtained^36^. Furthermore, given that an exploratory analysis has already been performed by the authors of the instrument^3^, we sought to confirm that proposal in the population of Brazilian students, by means of construct validity using CFA, an appropriate method for testing (confirming) whether the empirical structure observed in the set of items shows the same evidence as that of the theoretical construct of interest in the population analyzed^44^. 

Based on the results of the trend analysis and face validity carried out by four experts, the Arab study involving nursing students found that some items portrayed different intensities of incivility as compared to the original North American proposal; and in terms of construct validity, using Exploratory Factor Analysis, five factors were obtained for the scale with student behaviors. The study did not focus on the scale with items on faculty behaviors^30^. This aspect was addressed in a more recent study, using the same procedures, with 225 professors, which confirmed the 4-domain model for the scale dealing with faculty behaviors^45^. 

However, the Korean study, also conducted with students, identified that the scale with student behavior items had four factors by means of EFA, and that the faculty scale had two factors, similarly to the original North American theoretical proposal^14^. 

The path taken and the results described in our study indicate that the theoretical proposal^3^ is valid for the population studied, especially the existence of two domains (high level and low level of incivility) and the relevance of all the items attributed to faculty behaviors. 

When analyzing the factor loadings of the items in each domain of faculty behaviors, all the items were found to have high loadings contributing to the composition of the domains. On the other hand, when examining the items that portray student behaviors, three items do not reach 0.30, indicating their exclusion^46^. But for two of them, items 5 and 15, in both CFAs, with and without item 1, the statistical results show CI with upper limits greater than 0.30, and we chose to maintain both. It should also be emphasized that, when there is a theoretical justification, an item with a low factor loading can be maintained or reformulated^46^.

However, the low factor weights observed, at the cut-off point of 0.30, may be associated with the size of the sample in question, so the maintenance of those items, 5 and 15, in the Brazilian version for students requires further studies with different samples. 

The measures of fit of the model tested for student behavior (respectively, CFI = 1.000; 1.00; TLI = 1.000; NFI = 1.000; 1.000; RMSEA = 0.092; SRMR = 0.107) showed that all the indices indicate an acceptable fit, except for SRMR. As for faculty members, all the indices were acceptable. 

Among the frequent behaviors reported in the literature^3^ and in agreement with the theoretical statement^25^ ^),(^ ^28^ are those contained in the items that showed low factor loading in this study. It is noteworthy that an North American study, carried out during the pandemic, involving 675 undergraduate students and 35 nursing professors^39^, found that items 1 and 5 were the most frequent; a similar result was observed in a study involving 155 students and 40 professors in Oman^47^.

The behaviors related to items 1, 5 and 15, belonging to the Low Incivility Domain, were perceived as uncivil by the participants in this study, with the following means, considering scores ranging from 1 to 4: item 1 ( x = 2.76); item 5 ( x = 3.12); and item 15 ( x = 2,21). Also, when we asked about the frequency of their occurrence, as measured by the same parameters, the following means were found: item 1 ( x = 3.06); item 5 ( x = 3.40) and item 15 ( x = 2.27). 

These results show that students observe such behaviors relatively frequently, which supports their maintenance on the scale, since they are generally considered uncivilized based on the previously mentioned studies.

As for using a cell phone or another device for purposes unrelated to the class activity (item 5), which is considered to be disrespectful to others^28^, although the students perceived it as uncivil, they remained in contact with other people or websites, which is in line with the results of other studies carried out in different cultures^30^ ^),(^ ^48^ ^)-(^ ^49^. A recent study on student behavior, conducted in Canada with faculty members, also shows that such behavior is considered to be disruptive (for approximately 80% of the participants) and one of the most frequent (for approximately 60% of the participants)^50^. In our study, 54.5% of the students considered that it occurs frequently, and 33.3% that it occurs sometimes. As for the incivility level, it was predominantly considered to be moderately uncivil (46.2%) or highly uncivil (35.6%). These aspects reinforce the maintenance of this item in this study. 

With regard to item 15 (demanding new tests and deadline extensions), which is common in situations of disinterest when students are unprepared for school activities^28^, the impact of the dynamics of the remote activities employed during the period when this study was carried out may have interfered with students’ expectations given that learning situations are subject to change over time and may be more flexible, especially during the pandemic. The item refers to demanding something or not accepting a negative reply to a special favor requested and, in this context, it is uncivil behavior. This item, considered by the majority of students to display a low level of incivility (27.3% not uncivil and 37.9% not very uncivil), was reported as occurring rarely (33.7%), never (26.5%), sometimes (25.8%) or often (14%). 

Among the studies that analyzed the effects of the pandemic on nursing education, one pointed out that unrealistic expectations and communication difficulties negatively interfered with learning during that period, with discouragement and feelings of inadequacy^42^; another mentioned that online learning at that time was stressful and related to low satisfaction in learning^51^. Furthermore, the aforementioned study found a correlation between increased stress and uncivil behavior during that period^39^.

This context may also have interfered with the answers given, the relationship between professors and students and the motivation to attend or participate in educational activities, all of which are considered to be distracting or disrespectful in the classroom^28^ and are portrayed in the results of this study through the answers to item 1 of the survey (item 1- expressing disinterest, boredom or apathy towards the course content or subject). For students, this item is observed predominantly sometimes (43.2%) or often (33.7%), and it is primarily considered to be moderately uncivil (47.3%) or highly uncivil (18.6%), reinforcing the relevance of its inclusion in the instrument. These aspects indicate the existence of the behavior and the perception of the level of incivility related to the item for students in the sample.

The two studies that analyzed the construct validity for students used exploratory factor analysis because they considered the lack of validity studies in the Arab culture, as well as the possible cultural influence on the instrument^30^, or because they did not confirm the originally proposed two-domain model for the Korean population^14^. The two studies maintained all the items for both faculty and students.

Another measure applied to the instrument, with its original item structure, was the assessment of internal consistency, which proved to be highly reliable for the sample studied. The values obtained for student behavior (α = 0.937) and faculty behavior (α = 0.973) are considered to be an almost perfect correlation. The values obtained in the North American study were greater than 0.96 for students and 0.98 for faculty members^3^. In the Arab study^30^, alpha was 0.877, and in the Korean study, 0.940 for the student items related to incivility level^14^. It can be stated that the adapted instrument - Brazilian version showed internal consistency for its low and high incivility domains for student and faculty behaviors, with high values also found for ordinal alpha and McDonald’s omega.

In terms of reproducibility, it can be stated that the instrument showed adequate reproducibility, which was confirmed after 15 days with 29 students. The number of participants can be considered satisfactory for this analysis, given that a sample size of n = 20 can already be considered sufficient to obtain an adequate ICC result^52^.

When carrying out the test-retest with 10 students, the authors of the Korean study obtained a stability coefficient of 0.73 for the level of student incivility, and 0.64 for the level of faculty incivility^14^. In our study, the items relating to students showed good stability (ICC = 0.612), and those relating to faculty members showed moderate stability (ICC = 0.598). As for the stability of the items depicting the frequency of uncivil behaviors observed in the past 12 months, it was considered to be moderate for students (ICC = 0.474), and good for professors (ICC = 0.614).

Although this study was only carried out with students, the perception of the presence of incivility in the academic environment merits reflection, reinforcing the need to implement processes that encourage interpersonal relationships and dialogue regarding its existence, ways of dealing with it and, above all, as pointed out in a study^28^, the acceptance of responsibility on both sides. When faculty and students seek to build a more respectful educational environment, the result is increased civility^28^. Successful educational experiences that encourage a culture of civility, including the use of apps, have been reported^53^.

With regard to the relevance of the study, it is noteworthy that the use of the INE-R - Brazilian version for the population studied is pertinent, considering the reliability and stability of the survey, the full adequacy to the theoretical proposal of the original instrument in terms of the number of domains, the items related to faculty behaviors in their entirety and partially in relation to the items related to student behaviors. The availability of a validated survey in the context studied contributes to the science of nursing by making it possible to identify such a phenomenon or compare the results at an international level.

Finally, the perception of the students participating in this study regarding the existence of the behaviors addressed in the instrument under analysis reinforces the relevance of using the INE-R - Brazilian version to diagnose and intervene in this reality, in the different educational environments. The negative correlation between incivility behaviors experienced by students and values attributed to the profession, identified in an Iranian study, adds to this concern^54^.

We sought to carry out this study by following the recommendations for studies of this nature; however, limitations were identified, such as the characteristic of the sample’s being one of convenience, from a single public institution, and the number of participants, since it prevented the analysis of data invariance in random samples or discriminating groups. This number and the long collection time may be due to the pandemic occurring during the study. It should be noted that the inclusion criterion for the study required that students should have experienced the school environment for one year. For some, this period predominantly involved remote activities, which may have increased the perception of the level and frequency of uncivil acts. 

It is known that the process of validating a scale for different cultures is long and requires testing in various contexts^55^. Given this, it is hoped that this structure will be further analyzed psychometrically in new studies, with larger and more generalizable samples for the Brazilian reality, and then a decision can be made on whether or not to exclude items 5 and 15, which are found in the student version. This study was only carried out with students; validating the instrument with faculty members is required if there is any interest in using it with that population.

## Conclusion

The INE-R Survey - Brazilian version has been validated for the context studied, with reliability, as described by ICC, Cronbach’s alpha, ordinal alpha and McDonald’s omega, and construct validity as shown by CFA, with the model fitted for two domains, high and low incivility, as specified in its original version. All the items concerning faculty behaviors were confirmed in relation to the theoretical model. For the items related to student behaviors, the results point to the exclusion, for the time being, of item 1 and the suggestion of new psychometric tests of the student version, without that item, in different contexts.

The INE-R Survey - Brazilian version can be used to support studies and strategies that require measuring the perception or frequency of uncivil behavior in nursing higher education.
