# Supplementary material for: Psychometric properties of the Incivility in Nursing Education - Revised Survey - Brazilian version
Source: Rev Lat Am Enfermagem. 2024 Aug 12;32:e4215. doi: 10.1590/1518-8345.6950.4215 (PMC11321186; doi:10.1590/1518-8345.6950.4215)
Supplement: Translation

# Translation: **Propriedades psicométricas do *Incivility in Nursing Education - Revised Survey* versão brasileira** *

**DOI:** 10.1590/1518-8345.6950.4216

**Authors:** Vanessa dos Santos Ribeiro, Cynthia M. Clark, Claudia Benedita dos Santos, João Marôco, Jonas Bodini Alonso, Aline Helena Appoloni Eduardo, Emilia Campos de Carvalho

**Affiliations:** 1 Universidade de São Paulo, Escola de Enfermagem de Ribeirão Preto, Centro Colaborador da OPAS/OMS para o Desenvolvimento da Pesquisa em Enfermagem, Ribeirão Preto, SP, Brasil.; 2 Bolsista da Coordenação de Aperfeiçoamento de Pessoal de Nível Superior (CAPES), Brasil.; 3 Boise State University, School of Nursing, Boise, Idaho, ID, Estados Unidos da América.; 4 Bolsista do Conselho Nacional de Desenvolvimento Científico e Tecnológico (CNPq), Brasil.; 5 William James Center for Research, ISPA - Instituto Universitário, Lisboa, Portugal.; 6 Universidade Federal de São Carlos, Departamento de Enfermagem, São Carlos, SP, Brasil.

**Keywords:** Enfermagem, Incivilidade, Estudos de Validação, Educação, Estudantes de enfermagem, Professores

## Abstract

**Objetivo:**

analisar as propriedades psicométricas da versão brasileira do *Incivility in Nursing Education - Revised survey* com estudantes de graduação em enfermagem.

**Método:**

estudo metodológico, realizado em uma escola de enfermagem paulista. Trata-se da análise das propriedades psicométricas (fidedignidade e validade de construto) dos itens do INE-R *survey* - versão brasileira. A validade de construto foi realizada por Análise Fatorial Confirmatória e a fidedignidade pelo teste-reteste, para verificação da estabilidade do instrumento, calculada pelo Coeficiente de Correlação Intraclasse e por meio da consistência interna dos itens, segundo os coeficientes alfa de Cronbach, alfa ordinal e ômega de McDonald*.*

**Resultados:**

houve ajuste ao modelo proposto da análise fatorial confirmatória com dois fatores (baixa e alta incivilidade), com sugestão de exclusão de um item para estudantes. A maioria dos valores de ajustes para os itens dos estudantes e a totalidade para os professores atenderam às referências estabelecidas pela literatura; os valores para os Coeficientes de Consistência Interna foram maiores que 0,80 e os Coeficientes de Correlação Intraclasse, maiores que 0,75.

**Conclusão:**

a versão brasileira do *Incivility in Nursing Education - Revised Survey* encontra-se validada para o contexto estudado, apresentando fidedignidade satisfatória e validade, pela análise fatorial, que confirmou o modelo original com dois fatores, com 23 itens retratando comportamentos de estudantes e 24, de professores.

## Destaques

**(1)** A incivilidade é um fenômeno global e crescente no ensino superior.

**(2)** O ambiente incivil interfere na aprendizagem e nos indicadores de saúde.

**(3)** Apresenta-se um *survey* validado para medir a incivilidade no ensino de enfermagem.

## Introdução

Por mais de quatro décadas, o ambiente do ensino superior, incluindo-se o da Enfermagem, tem sido cenário de comportamento incivil, isto é, comportamento que não atende ao cumprimento de regras sociais para a convivência social^1^ *.* Incivilidade é qualquer comportamento que atrapalhe o processo de aprendizagem e interfira no ambiente de aprendizagem cooperativa. 

Algumas vezes descrita como abuso do poder sobre outros^2^, a incivilidade pode ser expressa por comportamentos direcionados a outra pessoa, desde formas menos agressivas, como insultos, desacordos e conflitos, ou por formas mais sérias de comportamentos agressivos, tal como a violência física entre indivíduos. A incivilidade pode também representar qualquer diálogo, interação ou atitude que atinge desfavoravelmente o bem-estar de estudantes ou docentes, enfraquecendo as relações profissionais, bloqueando o processo de ensino-aprendizagem e interferindo na qualidade da saúde física e mental^3^.

Se permitidos, os comportamentos incivis podem suscitar um processo em cadeia, no qual geram-se cada vez mais incivilidades. Nesse sentido, professores, tutores e gestores acadêmicos devem ser aptos a reduzir esses comportamentos nos distintos ambientes educacionais, evitando que incivilidades impactem negativamente as relações e o aprendizado, tanto teórico como prático, comprometendo a formação do futuro enfermeiro^4^ ^)-(^ ^5^.

A presença de incivilidade, tanto no ambiente de ensino como no de prática de enfermagem, é um fenômeno global^6^, constatado por estudos realizados em distintas culturas, como no Irã^7^ ^)-(^ ^8^, Emirados Árabes^6^, Afeganistão^9^, Canadá^10^, Itália^11^, China^12^, Indonésia^13^, Coreia^14^, Turquia^15^ e Estados Unidos da América^16^ ^)-(^ ^17^, dentre outros. Embora global, um estudo multicêntrico recente, com docentes de enfermagem de 10 países, identificou ser este um fenômeno percebido diferentemente entre os países que participaram do estudo^18^. 

Os atos incivis, ou outras formas de agressão, ocorrem tanto nas relações presenciais como nas relações virtuais, que frequentemente tratam das comunicações em redes sociais ou atividades acadêmicas remotas. Na enfermagem, observou-se o crescimento de comportamentos incivis no ensino *online* ^19^. Trata-se de um processo complexo e multidirecional em que estudantes, professores e preceptores podem contribuir para um clima de desrespeito, um pelo outro ou pelo processo de aprendizagem, empregando comportamentos relacionados ao gênero^20^, o não comparecimento às aulas^21^, respostas desnecessárias^22^ e atos impulsivos e agressivos exibidos *online* ^23^, dentre outros. 

Estudos apontam a correlação positiva entre estresse e comportamentos de incivilidade^4^ ^),(^ ^23^ ^)-(^ ^24^. Na vigência da pandemia de COVID-19, um período em que aulas remotas foram potencializadas, pesquisas reportaram o aumento de estresse, ansiedade e depressão referidos por estudantes de enfermagem^16^.

Como destacado no modelo conceitual de incivilidade em educação de enfermagem^25^, a partir da percepção da presença de comportamentos incivis, sobretudo de professores, os estudantes respondem com a possibilidade de se manterem na instituição e se conformarem conforme esperado (*loyalty*), de desafiarem o *status quo* e se esforçarem para mudar tais comportamentos na instituição (*voice*), ou de deixarem a escola (*exit*). As respostas emocionais, expectativas e decisões são dependentes do suporte que o estudante venha a receber, o que contribuirá para o sucesso da conclusão do curso^25^. 

*As* quebras das normas acordadas para o ambiente escolar, presencial ou remoto, pelos estudantes ou professores, agridem o processo de ensino-aprendizagem e devem ser identificadas, assim como seus fatores causais, para que possam ser manejadas e enfrentadas.

Um dos instrumentos criados para medir comportamentos não civis é o *Incivility in Nursing Education Survey* (INE). Ao construir o INE e em suas sucessivas revisões, a autora buscou identificar as percepções de estudantes e profissionais sobre os comportamentos que representariam a amplitude do fenômeno incivilidade. Estudos desenvolvidos em distintas culturas ou com profissionais e estudantes de diferentes tipos de programas de enfermagem apontam similaridades nesses comportamentos. Evidências usadas para desenvolver e revisar o INE-R^3^ descrevem a base para inclusão de comportamentos específicos de estudantes e professores. Tais comportamentos retratam um *continuum*, denominado *Continuum of Workplace Aggression* ^26^, que caracteriza uma estrutura confiável com uma série de comportamentos incivis, compreendendo desde comportamentos disruptivos ou de baixo nível de incivilidade até comportamentos mais sérios, como violência física ou tragédias. Quando relacionados aos estudantes, tais comportamentos contemplam distrações ou desrespeitos em sala de aula, desrespeito pelo outro ou, ainda, desinteresse geral pela aula^27^. 

O INE-R, desenvolvido em 2004, é composto pela definição de incivilidade, por dados de identificação do respondente, por um conjunto de itens em que constam os comportamentos incivis praticadas por professores e por outro conjunto de itens com comportamentos incivis praticados pelos estudantes, além de questões avaliativas. O processo de elaboração do INE e os de suas revisões estão descritos na literatura^28^ ^)-(^ ^29^. Em 2014 o INE-R original foi revisado e renomeado como *Incivility in Nursing Education - Revised* (INE-R) *Survey*, no qual alguns itens foram revisados e duas questões adicionadas com espaços em branco para o registro discursivo sobre as experiências dos respondentes com incivilidade acadêmica e modos de prevenir e enfrentar o problema. A versão INE-R foi validada entre estudantes e professores de enfermagem norte-americanos e obteve resultados adequados de sua estrutura e fidedignidade^3^ ^),(^ ^7^. 

O INE-R^3^ foi também validado com estudantes coreanos^14^ e árabes^30^ e, diante dos êxitos das investigações que o empregaram e da importância de se ter instrumento válido e confiável para se medir comportamentos incivis, figurou como objeto do presente estudo, cujo objetivo foi analisar as propriedades psicométricas da versão brasileira do *Incivility in Nursing Education - Revised Survey* com estudantes de graduação em enfermagem.

## Método

### Tipo de estudo, local e período

Estudo metodológico, referente às etapas de verificação da validade segundo análise da estrutura fatorial e da fidedignidade, realizado em uma instituição pública de ensino superior em enfermagem de uma cidade do interior do estado de São Paulo, Brasil, de junho de 2021 a março de 2022. Preliminarmente à etapa do estudo ora descrita, procedeu-se à adaptação cultural e a avaliação semântica do INE-R^31^. 

### Participantes e critérios de seleção

Foram convidados para participar do estudo todos os estudantes de graduação (n= 440) em enfermagem regularmente matriculados na instituição, exceto os do primeiro ano letivo, considerando-se que os participantes deveriam ter previamente pelo menos 12 meses de vivência no curso para responderem ao INE-R^3^. Dos elegíveis, isto é, todos estudantes do curso de graduação, exceto os do primeiro ano, 60% aceitaram participar do estudo. Não houve solicitação de desistência durante o preenchimento do instrumento.

### Coleta de dados e instrumentos utilizados

O recrutamento e a coleta de dados foram realizados após a aquiescência da instituição e do colegiado responsável pelo ensino de graduação. O convite e o fornecimento do consentimento informado e dos instrumentos de coleta foram feitos por uma das autoras a cada estudante, durante os intervalos das atividades acadêmicas. Nessas ocasiões, foram informados de que poderiam ser sorteados para a fase de pós-teste, prevista para ocorrer aproximadamente em 15 dias, conforme recomendações da literatura^32^.

Na sequência, cada estudante preencheu o Termo de Consentimento Livre e Esclarecido e, a seguir, o INE-R *Survey* - versão brasileira, individualmente, na presença do pesquisador. Para a análise da estabilidade do instrumento, 60 estudantes foram sorteados para responderem novamente o *survey*. 

O original INE-R *Survey* ^3^ ^)^ é autoaplicável, organizado em seções, iniciando pela conceituação de incivilidade e de ambiente acadêmico, seguindo-se dos dados demográficos do respondente (estudante ou professor), que serão determinados a critério do estudo em desenvolvimento, tais como gênero, idade, origem étnica/racial, anos de formação, programa ao qual o respondente pertence, posição/cargo do docente ou semestre letivo do estudante, dentre outros. No presente estudo foram considerados a idade, as identidades de gênero e étnico-racial e o semestre letivo do respondente.

A seção seguinte apresenta uma lista de 48 itens divididos em dois grupos, sendo um com 24 itens relativos a comportamentos de estudantes e outro com 24 itens relacionados a comportamentos de professores. Ambos apresentam dois fatores - alta incivilidade e baixa incivilidade - compostos por nove e quinze itens, respectivamente. Cada item é avaliado por uma escala Likert que corresponde ao nível de incivilidade que o comportamento representa ao respondente, com alternativas entre 1 e 4, a saber: não incivil; pouco incivil, moderadamente incivil e altamente incivil. Cada item também é avaliado quanto à frequência do comportamento nos últimos 12 meses, com quatro alternativas de respostas (escala de 1 a 4) correspondendo respectivamente a: nunca; raramente; às vezes e frequentemente. Duas questões completam a segunda sessão, abordando como o respondente percebe a magnitude do fenômeno na instituição (avaliada dentre quatro alternativas, desde não é problema até ser problema muito sério) e como cada respondente percebe a probabilidade de participação de professores e estudantes em atos incivis (avaliada com as alternativas: muito maior de professor, maior de professor, ambos, maior de aluno e muito maior de alunos)^3^. 

A última seção apresenta um conjunto de questões e um espaço narrativo, para o respondente descrever situações, opinar sobre incivilidade na educação em enfermagem, comportamentos incivis de estudantes e professores, nível de civilidade na instituição, priorização de estratégias para elevar nível de civilidade, bem como causa e consequência de incivilidade acadêmica^3^.

### Tratamento e análise dos dados

Os dados das diferentes variáveis foram inseridos, em dupla digitação, em uma planilha eletrônica do aplicativo Excel, constituindo um banco de dados estruturado. Para as variáveis semestre letivo, etnia e gênero, utilizou-se estatística descritiva; para a variável idade, como medida de tendência central, foram estabelecidas a média e a mediana, e como medida de dispersão, o desvio-padrão.

Foram realizadas as análises das propriedades psicométricas (fidedignidade e validade de construto) dos itens do INE-R *Survey* - versão brasileira. Preliminarmente, a normalidade dos dados foi verificada pelos valores absolutos de assimetria (Sk) e de curtose (Ku)^33^. A validade de construto foi realizada por Análise Fatorial Confirmatória e a fidedignidade pelo teste-reteste, para verificação da estabilidade do instrumento, calculada pelo Coeficiente de Correlação Intraclasse (ICC) e por meio da consistência interna dos itens, segundo coeficientes alfa de Cronbach, ordinal e ômega de McDonald^32^. A razão do número de participantes por número de itens do instrumento limitou outras análises.

A Análise Fatorial Confirmatória foi realizada pelo *software* R (R Core Team, 2021), versão 3.4.2, e uso do pacote *Latent Variable Analysis* (LAVAAN)^34^. Para estimação dos parâmetros foi utilizado o método de mínimos quadrados ponderados diagonalmente (*Diagonally Weighted Least Squares* - DWLS)^35^.

Em relação à análise da qualidade do ajuste do modelo, os índices de ajuste absoluto, índices de ajuste incremental e índices de discrepância populacional são tomados como alternativas para a verificação do ajuste^35^. O índice de discrepância populacional investigado foi a Raiz do Erro Quadrático Médio de Aproximação (*Root Mean Square Error of Approximation* - RMSEA), que estima se os parâmetros do modelo reproduzem a covariância populacional e nessas condições, o RMSEA tende a ser igual a zero. Também se avaliou a Raiz do Erro Quadrático Médio Residual (*Standardized Root Mean Square Residual* - SRMR), ou seja, a diferença padronizada entre a correlação observada e a prevista. Valores inferiores a 0,10 indicam ajustamento aceitável para RMSEA e inferiores a 0,08 são considerados aceitáveis para SRMR^35^. 

Em relação aos índices de ajustes incrementais, foram utilizados o Índice de Ajuste Normalizado (*Normed Fit Index* - NFI), o Índice de Ajuste Comparativo (*Comparative Fit Index* - CFI) e o Índice de Tucker-Lewis (TLI). Os valores de NFI e CFI, assim como o TLI, podem estar entre zero e um e valores acima de 0,90 indicam um ajuste aceitável^35^.

Para analisar a confiabilidade, utilizou-se a consistência interna determinada pelo coeficiente alfa (α) de Cronbach. Tal indicador permitirá comparar os resultados obtidos com os demais estudos que validaram o instrumento. Complementarmente, também foram analisados os valores dos coeficientes alfa ordinal e ômega de McDonald (ω), para cada domínio do instrumento (alto e baixo nível de incivilidade) em cada grupo de itens (os relativos a comportamentos de estudantes e os de professores). Os valores de α de Cronbach, de alfa ordinal e ômega de McDonald variam entre zero e um e, neste estudo, valores iguais a ou maiores que 0,70 foram considerados aceitáveis^32^. 

Para verificação da estabilidade da medida, procedeu-se ao teste-reteste com obtenção do Coeficiente de Correlação Intraclasse (ICC) à semelhança dos demais estudos que validaram esse instrumento. Nesta análise foi considerado o nível de significância de 5% (α= 0,05). Os valores do ICC entre 0,70 e 0,90 são considerados bons e os maiores que 0,90 são considerados excelentes^32^.

### Aspectos éticos

Os participantes foram convidados, sendo-lhes assegurada total privacidade de suas informações. A pesquisa somente foi iniciada após a aprovação da Comissão de Ensino e do Comitê de Ética em Pesquisa com Seres Humanos (CEP), ambos da instituição na qual o estudo foi realizado (Parecer CEP: 3.635.814 e Certificado de Apresentação de Apreciação Ética-CAAE 18508919.4.0000.5393).

## Resultados

Participaram do estudo 264 estudantes. Em relação às identidades de gênero e étnico-racial, 222 (84,1%) identificaram-se como mulheres, 40 (15,2%) como homens, um (0,4%) como ambos e outro (0,4%) como nenhum; 180 (68,2%) declararam-se brancos, 57 (21,6%) pardos, 22 (8,3%) pretos e 4 (1,5%) amarelos, enquanto 01 (0.4%) denominou-se indígena. Dos estudantes, 177 (67,0%) eram dos anos intermediários (terceiro ao sexto semestre letivo) e 87 (33,0%) eram dos anos concluintes (sétimo semestre em diante).

Os valores médios, respectivos desvios-padrão, assimetria (Sk) e curtose (Ku) dos níveis de incivilidade de comportamentos quando emitidos por estudantes e por professores, segundo os participantes (n = 264), estão apresentados na Tabela 1. Ao se considerarem os valores referentes à assimetria e curtose de cada item, estes são inferiores a três e sete, respectivamente, para todos os itens, comprovando a não violação da normalidade, pressuposto para realização da AFC.

**Tabela 1:** Estatística descritiva (médias, desvio-padrão, assimetria e curtose) dos níveis de incivilidade de comportamentos quando emitidos por estudantes e por professores, segundo os participantes (n = 264). Interior do estado de São Paulo, Brasil, 2021-2022

Comportamentos	Item	Média	Desvio-padrão	Assimetria	Curtose
**Relacionados aos estudantes**	01	2,76	0,84	-0,33	-0,45
	02	3,17	1,11	-0,94	-0,65
	03	2,87	0,82	-0,37	-0,37
	04	2,21	0,93	0,40	-0,70
	05	3,12	0,82	-0,77	0,15
	06	2,64	0,86	-0,27	-0,57
	07	2,48	0,84	-0,08	-0,60
	08	2,58	0,85	-0,01	-0,65
	09	2,49	0,92	0,10	-0,85
	10	3,08	1,01	-0,73	-0,70
	11	3,02	1,08	-0,72	-0,85
	12	3,17	0,85	-0,81	-0,01
	13	3,31	0,93	-1,13	0,12
	14	3,43	1,10	-1,56	0,61
	15	2,21	1,00	0,40	-0,90
	16	3,14	1,10	-0,94	-0,57
	17	3,05	1,12	-0,77	-0,88
	18	2,54	0,95	-0,10	-0,93
	19	3,42	1,11	-1,56	0,63
	20	3,51	1,08	-1,80	1,33
	21	3,44	1,04	-1,58	0,87
	22	3,47	1,12	-1,69	0,91
	23	3,45	1,13	-1,63	0,75
	24	3,47	1,14	-1,69	0,88
**Relacionados aos professores**	01	3,28	0,86	-1,08	0,04
	02	3,49	0,95	-1,05	-0,08
	03	3,31	0,86	-1,48	1,42
	04	3,09	0,92	-1,84	1,91
	05	3,01	0,96	-0,73	-0,33
	06	2,87	0,85	-2,23	4,02
	07	2,60	0,94	-2,40	4,48
	08	3,26	0,96	-1,56	1,87
	09	3,22	0,99	-1,49	0,97
	10	3,49	0,78	-2,74	6,71
	11	3,53	0,95	-0,91	0,16
	12	3,01	0,94	-1,82	1,49
	13	3,64	0,78	-2,01	2,21
	14	3,66	0,82	-1,87	1,73
	15	3,46	0,81	-1,88	1,58
	16	3,40	0,96	-1,86	1,56
	17	3,73	0,71	-1,88	1,60
	18	3,21	0,85	-1,08	0,04
	19	3,51	1,05	-1,05	-0,08
	20	3,57	1,01	-1,48	1,42
	21	3,53	1,02	-1,84	1,91
	22	3,53	1,08	-0,73	-0,33
	23	3,52	1,07	-2,23	4,02
	24	3,53	1,07	-2,40	4,48

Ao se considerarem os domínios, as médias do nível de incivilidade dos comportamentos de estudantes foram: Domínio Baixa Incivilidade x = 2,76; dp = 0,53; Domínio Alta Incivilidade x = 3,39, dp = 0,97; e para os professores foram: Domínio Baixa Incivilidade x = 3,22; dp = 0,69; Domínio Alta Incivilidade x = 3,58; dp = 0,89. A mediana dos itens de incivilidade atribuídos aos estudantes para o Domínio Baixa Incivilidade foi 3 e para o Domínio Alta Incivilidade foi 4; e a atribuída aos comportamentos de docentes para o Domínio Baixa Incivilidade foi 3; e para o Domínio Alta Incivilidade foi 4.

Em relação à frequência de comportamentos incivis nos últimos 12 meses, as médias e respectivos desvios-padrão para os itens relativos a comportamentos de docentes foram: Domínio Baixa Incivilidade x = 2,04; dp = 0,49; Domínio Alta Incivilidade x = 1,46, dp = 0,35; e para os relativos aos dos estudantes foram Domínio Baixa Incivilidade x = 2,43, dp = 0,53; Domínio Alta Incivilidade x = 1,54, dp = 0,49. As medianas, valores mínimos e máximos para todos os itens foram iguais para estudante e professores (mediana = 2; mínimo = 1; máximo = 4), baixa e alta incivilidade.

Na AFC, dentre os comportamentos de estudantes, todos os itens do Domínio Alta Incivilidade apresentaram cargas fatoriais elevadas, variando de 0,734 a 0,999. Para o Domínio Baixa Incivilidade, três itens (3, 4 e 8) apresentaram cargas variando de 0,304 a 0,396 e os demais demonstraram valores variando de 0,613 a 0,825, exceto os itens 1, 5 e 15 que apresentaram carga fatorial < 0,300. O item 1- expressar desinteresse, tédio ou apatia sobre o conteúdo do curso ou assunto - apresentou carga fatorial 0,149, IC[95%]: [0,105; 0,192]; o item 5- usar computador, celular ou outros dispositivos eletrônicos durante uma aula, reunião ou atividade para fins não relacionados - apresentou carga fatorial igual a 0,290, IC[95%]: [0,249; 0,332] ; e o item 15- exigir provas substitutas, extensões de prazos ou outros favores especiais - apresentou carga fatorial de 0,299; IC[95%]: [0,259; 0,338] (Tabela 2).

**Tabela 2:** Resultados da Análise Fatorial Confirmatória em relação às cargas fatoriais dos itens referentes aos comportamentos de estudantes (n = 264). Interior do estado de São Paulo, Brasil, 2021-2022

Domínio	Item	Cargas fatoriais	Intervalo de 95% de confiança	Erro-padrão	Z*	*P*-valor
**Alta Incivilidade**	Item13	0,734	(0,702; 0,766)	0,039	18,916	0,000
	Item 14	0,946	(0,930; 0,962)	0,016	57,785	0,000
	Item17	0,855	(0,833; 0,877)	0,023	37,411	0,000
	Item 19	0,988	(0,983; 0,993)	0,004	236,035	0,000
	Item 20	0,999	(0,997; 1,000)	0,001	1431,998	0,000
	Item 21	0,958	(0,947; 0,969)	0,011	85,421	0,000
	Item 22	0,999	(0,997; 1,000)	0,001	941,880	0,000
	Item 23	0,999	(0,997; 1,000)	0,001	1210,095	0,000
	Item 24	0,999	(0,997; 1,000)	0,001	740,119	0,000
**Baixa Incivilidade**	Item 1	0,149	(0,105; 0,192)	0,070	2,119	0,000
	Item 2	0,802	(0,772; 0,831)	0,032	24,995	0,000
	Item 3	0,350	(0,311; 0,388)	0,060	5,861	0,000
	Item 4	0,304	(0,261; 0,346)	0,066	4,618	0,000
	Item 5	0,290	(0,249; 0,332)	0,062	4,653	0,000
	Item 6	0,641	(0,604; 0,678)	0,043	15,021	0,000
	Item 7	0,538	(0,500; 0,576)	0,048	11,188	0,000
	Item 8	0,396	(0,354; 0,438)	0,060	6,643	0,000
	Item 9	0,577	(0,537; 0,616)	0,050	11,587	0,000
	Item 10	0,825	(0,798; 0,853)	0,028	29,948	0,000
	Item 11	0,825	(0,798; 0,852)	0,030	27,392	0,000
	Item 12	0,758	(0,728; 0,788)	0,034	22,184	0,000
	Item 15	0,299	(0,259; 0,338)	0,062	4,801	0,000
	Item 16	0,776	(0,746; 0,805)	0,036	21,609	0,000
	Item 18	0,613	(0,581; 0,644)	0,041	14,809	0,000

*Z = Variável normal padronizada

Quanto aos comportamentos de professores, a menor das cargas fatoriais dos itens foi 0,596 (item 18), que pertence ao Domínio Baixa Incivilidade, e as maiores (itens 20, 22, 23 e 24) pertencem ao Domínio Alta Incivilidade; portanto, todos os itens tiveram cargas fatoriais satisfatórias (Tabela 3).

**Tabela 3:** Resultados da Análise Fatorial Confirmatória em relação às cargas fatoriais dos itens relativos a comportamentos de professores (n = 264). Interior do estado de São Paulo, Brasil, 2021-2022

Domínio	Item	Cargas fatoriais	Intervalo de 95% de confiança	Erro-padrão	Z*	***P-*valor**
**Alta Incivilidade**	Item13	0,888	(0,870; 0,906)	0,026	33,940	0,000
	Item 14	0,963	(0,952; 0,975)	0,013	73,082	0,000
	Item17	0,948	(0,935; 0,961)	0,016	59,428	0,000
	Item 19	0,990	(0,985; 0,995)	0,004	241,701	0,000
	Item 20	0,997	(0,995; 0,999)	0,002	606,938	0,000
	Item 21	0,993	(0,989; 0,996)	0,003	313,800	0,000
	Item 22	1,000	(1,000; 1,000)	0,001	1203,589	0,000
	Item 23	0,998	(0,995; 1,000)	0,001	729,320	0,000
	Item 24	0,998	(0,996; 1,000)	0,001	739,529	0,000
**Baixa Incivilidade**	Item 1	0,827	(0,807; 0,848)	0,026	31,722	0,000
	Item 2	0,930	(0,914; 0,946)	0,020	47,166	0,000
	Item 3	0,674	(0,647; 0,701)	0,043	15,696	0,000
	Item 4	0,816	(0,706; 0,836)	0,029	28,355	0,000
	Item 5	0,807	(0,786; 0,827)	0,024	33,571	0,000
	Item 6	0,762	(0,739; 0,785)	0,028	26,902	0,000
	Item 7	0,709	(0,684; 0,734)	0,036	19,748	0,000
	Item 8	0,899	(0,882; 0,917)	0,018	51,310	0,000
	Item 9	0,891	(0,873; 0,909)	0,020	44,766	0,000
	Item 10	0,779	(0,756; 0,803)	0,033	23,809	0,000
	Item 11	0,937	(0,921; 0,954)	0,017	54,505	0,000
	Item 12	0,814	(0,794; 0,834)	0,026	31,101	0,000
	Item 15	0,782	(0,758; 0,805)	0,034	23,185	0,000
	Item 16	0,900	(0,883; 0,917)	0,020	45,231	0,000
	Item 18	0,596	(0,567; 0,625)	0,050	12,002	0,000

*Z = Variável normal padronizada

A partir das estimativas pontuais e por intervalos das cargas fatoriais dos itens da versão brasileira do instrumento, sugere-se uma organização estrutural dos itens com dois fatores, alta incivilidade e baixa incivilidade, semelhante à estrutura original, com a exclusão do item 1 para os estudantes, que apresentou IC95% para a carga fatorial incluindo somente valores inferiores a 0,30.

Os resultados da Análise Fatorial Confirmatória em relação às cargas fatoriais dos itens relativos a comportamentos de estudantes, com a exclusão do item 1 para estudantes, estão apresentados na Tabela 4.

**Tabela 4:** Resultados da Análise Fatorial Confirmatória em relação às cargas fatoriais dos itens relativos a comportamentos de estudantes (n = 264). Interior do estado de São Paulo, Brasil, 2021-2022

Domínio	Item	Cargas fatoriais	Intervalo de 95% de confiança	Erro-padrão	Z*	***P-*valor**
**Alta Incivilidade**	Item13	0,734	(0,702; 0,766)	0,039	18,865	0,000
	Item 14	0,946	(0,930; 0,962)	0,016	57,844	0,000
	Item17	0,855	(0,834; 0,877)	0,023	37,411	0,000
	Item 19	0,988	(0,983; 0,993)	0,004	236,087	0,000
	Item 20	0,999	(0,997; 1,000)	0,001	1431,743	0,000
	Item 21	0,958	(0,947; 0,969)	0,011	85,376	0,000
	Item 22	0,999	(0,997; 1,000)	0,001	941,885	0,000
	Item 23	0,999	(0,997; 1,000)	0,001	1210,134	0,000
	Item 24	0,999	(0,997; 1,000)	0,001	740,191	0,000
**Baixa Incivilidade**	Item 2	0,801	(0,771; 0,830)	0,032	24,968	0,000
	Item 3	0,346	(0,307; 0,384)	0,060	5,768	0,000
	Item 4	0,301	(0,258; 0,343)	0,066	4,565	0,000
	Item 5	0,287	(0,246; 0,328)	0,063	4,579	0,000
	Item 6	0,640	(0,604; 0,677)	0,043	14,967	0,000
	Item 7	0,535	(0,497; 0,572)	0,048	11,058	0,000
	Item 8	0,394	(0,352; 0,436)	0,060	6,579	0,000
	Item 9	0,575	(0,536; 0,615)	0,050	11,535	0,000
	Item 10	0,825	(0,798; 0,852)	0,028	29,942	0,000
	Item 11	0,824	(0,797; 0,852)	0,030	27,339	0,000
	Item 12	0,757	(0,728; 0,787)	0,034	22,143	0,000
	Item 15	0,299	(0,259; 0,338)	0,062	4,794	0,000
	Item 16	0,775	(0,746; 0,805)	0,036	21,579	0,000
	Item 18	0,612	(0,580; 0,644)	0,041	14,791	0,000

*Z = Variável normal padronizada

Frente aos resultados apresentados na Tabela 4, com a exclusão do item 1, e pelas justificativas anteriormente apresentadas, a opção foi pela manutenção de todos os demais 23 itens para os estudantes.

Para avaliação de construto, a partir da Análise Fatorial Confirmatória, as medidas adotadas de ajuste do modelo à população, testado para os itens que retratam comportamentos de estudantes e de professores, respectivamente, foram: Qui quadrado (χ2 = 815,609 e 491,440); Graus de Liberdade (GL= 229; 251); (χ2/GL = 3,56 e 1,96); p-valores (< 0,001; < 0,001); Índice de Ajuste Comparativo (CFI = 1,000; 1,000); Índice de Tucker-Lewis ou Índice de Ajuste Não Normalizado (TLI = 1,000; 1,000); Índice de Ajuste Normalizado (NFI =1,000; 1,000); Raiz do Erro Quadrático Médio de Aproximação (RMSEA = 0,092; 0,044); Intervalo com 95% de Confiança para RMSEA (0,083; 0,101) e (0,035; 0,055) e Raiz do Erro Quadrático Médio Residual (SRMR = 0,107; 0,053). A maioria dos índices para estudantes e todos os índices para os professores indicam um ajustamento aceitável.

As representações dos Domínios Baixa Incivilidade e Alta Incivilidade e de seus itens do INE-R - versão brasileira, com suas respectivas cargas fatoriais, da Análise Fatorial Confirmatória, estão apresentadas na Figura 1. A estrutura é apresentada para professores, conforme proposto no modelo original do instrumento, e para estudantes, respeitadas as justificativas constantes na discussão sobre os itens.

**Figura 1:** Diagramas de caminhos dos Domínios Baixa incivilidade e Alta incivilidade do *Incivility in Nursing Education - Revised Survey* versão brasileira, considerando-se a estrutura fatorial original. Interior do estado de São Paulo, Brasil, 2021-2022 *It =itens; ^†^BxInv = Baixo nível de incivilidade; ^‡^AltInv = Alto nível de incivilidade

A fidedignidade do INE-R *Survey* - versão brasileira foi avaliada pela consistência interna e pela reprodutibilidade. A consistência interna, avaliada pelos coeficientes Alfa de Cronbach, alfa Ordinal e ômega de McDonald, está apresentada na Tabela 5.

**Tabela 5:** Consistência interna do *Incivility in Nursing Education - Revised Survey* versão brasileira, segundo o grupo de comportamentos incivis, as dimensões de incivilidade e itens que os compõem (n = 264). Interior do estado de São Paulo, Brasil, 2021-2022

Grupo de Comportamentos	Dimensões de incivilidade	Itens	Valor de Alfa Cronbach (IC 95%)	Valor Alfa ordinal	Valor de Ômega
**Estudante**	Alta	13, 14, 17, 19, 20, 21, 22, 23, 24	0,966 (0,962; 0,974)	0,984	0,976
	Baixa	2 a 12, 15, 16, 18	0,853 (0,827; 0,879)	0,880	0,857
**Professor**	Alta	13, 14, 17, 19, 20, 21, 22, 23, 24	0,977 (0,973; 0,981)	0,993	0,989
	Baixa	1 a 12, 15, 16, 18	0,949 (0,940; 0,958)	0,964	0,951

A consistência interna pode ser considerada altamente satisfatória tanto para os itens que avaliam os comportamentos dos estudantes como para os dos professores, para ambos os domínios de incivilidade, evidenciando adequada fidedignidade na amostra estudada. 

Quanto à análise da reprodutibilidade do instrumento, dos 60 estudantes sorteados para essa fase, somente 29 participaram. Em relação ao nível de incivilidade a estabilidade, analisada pelo Coeficiente de Correlação Intraclasse, foi considerada com concordância boa com valor de ICC em relação aos itens para os comportamentos de estudantes (ICC = 0,612, IC [95%]: [0,330; 0,796] e *p*-valor < 0,0001) e moderada para os comportamentos dos professores (ICC = 0,598 IC[95%]:[0,301; 0,789] e *p*-valor < 0,0001).

Para a estabilidade dos itens que retratam a frequência dos comportamentos incivis observados nos últimos 12 meses, nas fases de pré- e pós-teste, a concordância foi considerada moderada para os estudantes (0,474, IC [95%]: [0,148; 0,710] e *p*-valor = 0,003) e boa para os professores (ICC = 0,615, IC [95%]: [0,332; 0,789] e *p*-valor < 0,0001). 

## Discussão

No presente estudo analisaram-se as propriedades psicométricas da versão brasileira do INE-R *Survey* ^3^. O INE-R *survey* tem sido útil para identificar as percepções de atos incivis de alunos e de professores de enfermagem^18^, permitindo estabelecer as distinções das percepções dos dois grupos^7^. 

Em relação ao instrumento, a variável do estudo é uma variável latente, mensurada por meio de opções de respostas em escala do tipo Likert, que variam de 1 a 4, portanto, não simétrica (inexiste ponto neutro equidistante dos extremos) e não homogênea em relação ao número de itens, pois cada domínio tem, **r**espectivamente, nove e 15 itens^36^. 

O período da coleta de dados deu-se na vigência da pandemia da COVID-19, ocasião em que as atividades de estudantes e de docentes se apresentavam em fase de ajustes, com introdução de ensino *online*. Tal situação retratou sobrecargas de demandas e dificuldades de acesso e manuseio de dispositivos^37^. Segundo a literatura, mudanças nas percepções de frequência ou nível de incivilidades de comportamentos de estudantes e professores foram identificadas no período de pandemia^38^ ^)-(^ ^39^.

No presente estudo, os itens correspondentes aos comportamentos de estudantes e de professores foram avaliados apenas por estudantes. O estudo original norte-americano validou o instrumento por professores e estudantes e para os conjuntos de itens de comportamentos de estudantes e de professores^3^. Os demais validaram o instrumento inteiro apenas com estudantes^14^ ^)^ ou somente os itens relativos aos comportamentos de estudantes^30^. O instrumento pode ser avaliados para cada um dos sujeitos-foco (estudantes ou professores) ou conjuntamente^3^.

As percepções referidas sobre comportamentos de professores, no presente estudo, dizem respeito às experiências com docentes de uma instituição pública, secular (não religiosa), que majoritariamente trabalham por longos períodos na mesma instituição. A literatura cita um estudo realizado na África do Sul^40^, em que o tempo de trabalho na mesma instituição apresentou associação com aumento da incivilidade, e um outro estudo que menciona haver níveis aumentados de incivilidade na academia^41^. Tais aspectos podem ter gerado impacto na expressão de comportamentos incivis.

Portanto, o tipo prolongado de vínculo do professor na instituição^40^, o fato de a coleta de dados ter ocorrido durante um período de ajustes a novas demandas impostas pela pandemia^16^ ^)^ e o aumento do uso de tecnologias remotas para as aulas^38^ ^),(^ ^42^ durante ou nos semestres anteriores à realização do estudo podem ter impactado a resposta dos estudantes, ou seja, a percepção de frequência ou do nível de comportamentos de incivilidades.

Em relação ao número de participantes, ao se empregar um instrumento como o descrito, há indicação para que, ao se realizar análise fatorial, sejam computados de 5 a 10 respondentes por questão do instrumento. Contudo, ao se usar um modelo de equações estruturais, há literatura que recomenda de 10 a 15 participantes por variável no modelo^36^. No presente estudo, o tamanho amostral de 264 atendeu à primeira condição, mas impossibilitou a análise da invariância fatorial do modelo. No estudo original, a amostra foi de 310 estudantes norte-americanos^3^; o estudo que validou o instrumento para a cultura coreana teve 284 participantes^14^ e a árabe, 389 participantes^30^. Todos empregaram análise fatorial em suas análises. 

Em relação à análise descritiva do instrumento em tela, optamos por apresentar a percepção dos participantes sobre o nível de incivilidade e a sua frequência por meio de mediana, ainda que tivéssemos descrito a média para efeito de comparação com os estudos que validaram ou empregaram esse instrumento em outras culturas^14^ ^),(^ ^30^. Tal indicador (média) pode não representar a variação da amostra com clareza por se tratar de escala tipo Likert, portanto, uma escala qualitativa ordinal, segundo o nível de mensuração^36^.

A título de comparação dos níveis de incivilidade, para os comportamentos de estudante, o presente estudo teve média geral igual a 3,0, enquanto nos estudos coreano e árabe, estas foram x = 3,11 e x = 3,45, respectivamente^14^ ^),(^ ^30^; e, para os comportamentos de professores, nossos resultados foram x = 3,35 e os do estudo coreano x = 3,17^14^. As médias das frequências de ocorrência dos comportamentos nos últimos 12 meses, referidas em nosso estudo para os comportamentos dos estudantes e de professores, foram 2,10 e 1,82, respectivamente, enquanto no estudo árabe a média geral para os itens dos estudantes foi 1,99^30^.

Ainda, a análise descritiva dos dados do presente estudo aponta aspectos que merecem considerações. Um deles diz respeito à distribuição das respostas. Foi identificado o efeito teto-piso (*ceiling*/*floor effect*) para vários itens do INE-R *Survey* - versão brasileira. Isso indica que a distribuição dos escores é assimétrica, ou seja, retrata a percentagem dos participantes que pontuaram os mais baixos ou elevados níveis da medida^32^. Em estudo que usou o INE, também se observou o efeito teto-piso, havendo mais de 15% de frequência para a percepção de comportamentos de estudantes e professores em ambos os polos da escala utilizada^29^.

Sabe-se que comportamentos disruptivos são mais prevalentes que os incivis de alta incivilidade^29^. E é mais provável verificarem-se comportamentos não verbais rudes e declarações de comentários humilhantes do que comportamentos ameaçadores ou atos violentos^3^.

A tendência identificada de os participantes atribuírem maior nível de incivilidade a um mesmo comportamento, quando este é realizado por professores do que quando realizado por estudantes, também já havia sido descrita em um estudo que analisou o efeito moderador da hierarquia social em comportamentos incivis no trabalho^43^ ^),^ mostrando que a incivilidade percebida foi maior entre os atos incivilizados perpetrados pelos gerentes em comparação com a incivilidade percebida se perpetrada por um colega.

Em relação à validade e à confiabilidade, a literatura aponta distintas alternativas para análise das propriedades psicométricas, a depender da natureza da variável, forma de medida, tamanho amostral e características dos dados obtidos^36^. Ainda, dado já existir uma análise exploratória feita pelas autoras do instrumento^3^, buscamos confirmar tal proposta na população de estudantes brasileiros, por meio da validade de construto empregando a AFC, uma técnica adequada para testar (confirmar) se a estrutura empírica observada no conjunto de itens apresenta as mesmas evidências do construto teórico de interesse na população analisada^44^. 

O estudo árabe com estudantes de enfermagem verificou, a partir dos resultados da análise de tendências e com validade de face realizada por quatro peritos, que alguns itens retratavam intensidades distintas de incivilidade em relação à proposta original norte-americana; e na validade de construto, por meio de Análise Fatorial Exploratória, obteve cinco fatores para a escala com comportamentos de estudantes. O estudo não teve como objeto a escala com itens sobre comportamentos de docentes^30^. Tal aspecto foi abordado em estudo mais recente, utilizando os mesmos procedimentos, com 225 professores, e que confirmou o modelo de 4 domínios para a escala que trata de comportamentos dos professores^45^. 

Já o estudo coreano, também com estudantes, identificou que a escala com itens de comportamentos de estudantes apresentava, por meio de AFE, quatro fatores, e que a de professores apresentava dois fatores à semelhança da proposta teórica original norte-americana^14^. 

O percurso percorrido e os resultados descritos em nosso estudo indicam ser a proposta teórica^3^ válida para a população estudada, em especial a existência de dois domínios (alto nível e baixo nível de incivilidade) e a pertinência de todos os itens atribuídos aos comportamentos de professores. 

Ao se analisar as cargas fatoriais dos itens em cada domínio de comportamentos de professores, identifica-se ajuste de todos os itens com cargas elevadas de contribuição para composição dos domínios. Em contrapartida, ao se examinar os itens que retratam os comportamentos de estudantes, três itens não alcançam o valor 0,30, indicando sua exclusão^46^. Mas, para dois deles, itens 5 e 15, em ambas AFCs, com e sem o item 1, os resultados estatísticos indicam IC com limites superiores maiores que 0,30 e a opção foi pela manutenção de ambos. Reforça-se ainda que, quando existe justificativa de ordem teórica, um item com baixa carga fatorial pode ser mantido ou reformulado^46^.

Entretanto, os baixos pesos fatoriais, no limite do ponto de corte de 0,30, observados podem estar associados ao tamanho da amostra em questão, pelo que, a manutenção desses itens, 5 e 15, na versão brasileira para estudantes necessita de mais estudos com amostras diferentes. 

Os dados das medidas de ajustes do modelo testado para os comportamentos de estudantes (respectivamente, CFI = 1,000; 1,00; TLI = 1,000; NFI = 1,000; 1,000; RMSEA = 0,092; SRMR = 0,107) evidenciaram que os todos os índices indicam um ajustamento aceitável, exceto o SRMR. Para os professores todos os índices foram aceitáveis. 

Dentre os comportamentos frequentes relatados na literatura^3^ ^)^ e concordantes ao enunciado teórico^25^ ^),(^ ^28^ encontram-se aqueles contidos nos itens que apresentaram baixa carga fatorial no presente estudo**.** Destaca-se que um estudo norte-americano, realizado durante a pandemia, com 675 estudantes de graduação e 35 professores de enfermagem^39^, identificou que os itens 1 e 5 foram os mais frequentes; igual resultado observou-se no estudo com 155 estudantes e 40 professores em Omã^47^.

Os comportamentos relacionados aos itens 1, 5 e 15, pertencentes ao domínio Baixa Incivilidade, foram percebidos como incivis pelos participantes do presente estudo, com as seguintes médias, considerando-se os escores variando de 1 a 4: item 1 ( x = 2,76); item 5 ( x = 3,12); e item 15 ( x = 2,21). Ainda, quando se perguntou sobre a frequência de sua ocorrência, medida pelos mesmos parâmetros, apresentaram-se as seguintes médias: item 1 ( x = 3,06); item 5 ( x = 3,40) e item 15 ( x = 2,27). 

Esses resultados mostram que os alunos observam tais comportamentos com relativa frequência, o que apoia a manutenção dos itens na escala, uma vez que eles são geralmente considerados incivilizados com base nos estudos anteriores referidos.

Quanto a usar um telefone celular ou outro dispositivo para fins não relacionados à atividade da aula (item 5), considerado como desrespeito para com o outro^28^, apesar de os estudantes perceberem o comportamento como incivil, permaneceram em contato com outras pessoas ou com *websites,* a exemplo de resultados de outros estudos realizados em diferentes culturas^30^ ^),(^ ^48^ ^)-(^ ^49^. Um estudo recente sobre comportamentos de estudantes, como o desenvolvido no Canadá com professores, também aponta ser esse comportamento considerado disruptivo (para cerca de 80% dos participantes) e um dos mais frequentes (para cerca de 60% dos participantes)^50^. Em nosso estudo, 54,5% dos estudantes consideraram que ele ocorre frequentemente e 33,3% que ele ocorre às vezes. Quanto ao nível de incivilidade, foi predominantemente considerado moderadamente incivil (46,2%) ou altamente incivil (35,6%). Tais aspectos reforçam a manutenção desse item no presente estudo. 

Em relação ao item 15 (exigir novas provas e ampliação de prazos), comum nas situações de desinteresse em que não se está preparado para as atividades escolares^28^, o impacto da dinâmica das atividades remotas empregadas no período de realização do presente estudo pode ter interferido nas expectativas dos alunos frente às situações de aprendizagem serem passiveis de mudança ao longo do tempo e poderem ser mais flexíveis, em especial durante a pandemia. O item refere-se a exigir algo ou a não aceitar uma resposta negativa a um favor especial solicitado e, nesse contexto, trata-se de comportamento incivil. Este item, considerado pela maioria dos estudantes como baixo nível de incivilidade (27,3% não incivil e 37,9% pouco incivil), foi relatado como ocorrendo raramente (33,7%), nunca (26,5%), às vezes (25,8%) ou frequentemente (14%). 

Dentre os estudos que analisaram os efeitos da pandemia no ensino de enfermagem, um apontou que as expectativas não realísticas e dificuldades de comunicação interferiram negativamente na aprendizagem nesse período, com desânimo e sentimento de inadequação^42^; outro mencionou ser o aprendizado *online* nesse período estressante e relacionado à baixa satisfação em aprender^51^. Ainda, o estudo já referido apontou correlação entre aumento do estresse e comportamentos incivis nesse período^39^.

Tal contexto também pode ter interferido nas respostas emitidas, nas relações entre professor e estudante e na motivação para assistir ou participar das atividades educacionais, aspectos considerados como distrações ou desrespeitos em sala de aula^28^ e retratados nos resultados do presente estudo por meio das respostas ao item 1 do *survey* (item 1- expressar desinteresse, tédio ou apatia sobre o conteúdo do curso ou assunto). Para os estudantes, esse item é observado predominantemente às vezes (43,2%) ou frequentemente (33,7%), e é considerado prioritariamente como moderadamente incivil (47,3%) ou altamente incivil (18,6%), reforçando a pertinência de sua inclusão no instrumento. Tais aspectos apontam a existência do comportamento e a percepção do nível de incivilidade relativo ao item para estudantes na amostra. 

Os dois estudos que analisaram a validade de construto de estudantes utilizaram a análise fatorial exploratória, por considerarem a inexistência de estudos de validade na cultura árabe, bem como a eventual influência cultural no instrumento^30^, ou por não confirmarem o modelo original proposto de dois domínios em população coreana^14^. Ambos mantiveram todos os itens, tanto para os professores como para estudantes.

Outra medida aplicada ao instrumento, com sua estrutura original de itens, foi a verificação da consistência interna que se mostrou altamente confiável para a amostra estudada. Os valores obtidos para os comportamentos de estudantes (α = 0,937) e de professores (α = 0,973) são considerados como correlação quase perfeita. Os valores obtidos no estudo norte-americano foram maiores que 0,96 para estudantes e 0,98 para professores^3^. No estudo árabe^30^, o alfa foi 0,877 e no coreano, 0,940 para os itens de estudantes relacionados ao nível de incivilidade^14^. Pode-se afirmar que o instrumento adaptado - versão brasileira apresentou consistência interna para seus domínios de baixa e alta incivilidade para os comportamentos de estudantes e de professores, com valores altos também encontrados para alfa ordinal e ômega de McDonald.

Em relação à reprodutividade, pode-se dizer que o instrumento apresentou reprodutibilidade adequada, verificada após 15 dias, com 29 estudantes. O número de participantes pode ser considerado aceito como satisfatório para tal análise, dado que uma amostra de tamanho n = 20 já pode ser considerada suficiente para obter um resultado de ICC adequado^52^.

Ao realizarem o teste-reteste com 10 alunos, os autores do estudo coreano obtiveram coeficiente de estabilidade 0,73 para nível de incivilidade de estudantes e 0,64 para o de professores^14^. Em nosso estudo, os itens referentes aos estudantes apresentaram estabilidade boa (ICC = 0,612) e os relativos aos professores, moderada (ICC = 0,598). Já, em relação à estabilidade dos itens que retratam a frequência dos comportamentos incivis observados nos últimos 12 meses, esta foi considerada moderada para os estudantes (ICC = 0,474) e boa, para os professores (ICC = 0,614).

Embora este estudo tenha sido desenvolvido apenas com estudantes, a percepção da presença do fenômeno incivilidade no ambiente acadêmico merece reflexão, reforça a necessidade de se implantarem processos que estimulem as relações interpessoais e o diálogo sobre tal existência, as formas de enfrentamento e, sobretudo, como apontado em um estudo^28^, a assunção da responsabilidade de ambas as partes. Quando professores e estudantes buscam construir ambiente educacional mais respeitável, o resultado é o crescimento da civilidade^28^. Experiências educativas exitosas, com est**í**mulo da cultura da civilidade, incluindo-se o uso de aplicativos, têm sido reportadas^53^.

Quanto à relevância do estudo, cabe destacar que há pertinência do uso do INE-R - versão brasileira para a população estudada, considerando a confiabilidade e estabilidade do *survey*, adequação integral à proposta teórica do instrumento original quanto ao número de domínios, aos itens relacionados com os comportamentos de professores em sua totalidade e parcialmente em relação aos itens relacionados aos comportamentos dos estudantes. A disponibilização de um *survey* validado no contexto estudado colabora com a ciência da Enfermagem ao permitir identificar tal fenômeno ou comparar os resultados em nível internacional**.**

Por fim, a percepção dos estudantes participantes deste estudo sobre a existência dos comportamentos abordados no instrumento em análise reforça a pertinência de se empregar o INE-R - versão brasileira para diagnosticar e intervir nessa realidade, nos diferentes ambientes do ensino. A correlação negativa entre comportamentos de incivilidade vividos por estudantes e valores atribuídos à profissão, identificada em um estudo iraniano, soma-se a essa preocupação^54^.

Buscou-se realizar a presente pesquisa observando-se as recomendações para estudos dessa natureza; contudo, foram identificadas limitações, como a característica da amostra de ser de conveniência, de uma única instituição pública, e o número de participantes, visto que este inviabilizou a análise da invariância dos dados em amostras aleatórias ou grupos discriminantes. Tal número e o longo tempo de coleta podem ser decorrentes da pandemia ocorrida durante a realização do estudo. Cabe lembrar que o critério de inclusão ao estudo exigia a vivência do estudante por um ano no ambiente escolar; para alguns, esse prazo deu-se predominantemente com atividades remotas, fato que pode ter potencializado a percepção do nível e da frequência de atos incivis. 

Sabe-se que o processo de validação de uma escala para culturas distintas é longo e demanda testes em vários contextos^55^; diante disso, espera-se que esta estrutura seja ainda submetida à análise psicométrica em novos estudos, com amostras de maior dimensão e mais generalizáveis para a realidade brasileira e, então, a decisão pela exclusão ou não dos itens 5 e 15, referidos na versão para estudantes poderá ser tomada.

Este estudo foi desenvolvido apenas com estudantes; torna-se necessário validar o instrumento com professores caso haja interesse em empregá-lo nessa população.

## Conclusão

O INE-R *Survey* - versão brasileira encontra-se validado para o contexto estudado, com fidedignidade, descrita pelo ICC, alfa de Cronbach, alfa ordinal e ômega de McDonald e validade de construto reiterada pela AFC, com o modelo ajustado para dois domínios, de alta e baixa incivilidade, como previsto em sua versão original. Em relação aos itens referentes aos comportamentos de professores, houve confirmação de todos em relação ao modelo teórico. Para os itens relacionados aos comportamentos de estudantes, os resultados apontam para a exclusão, por ora, do item 1 e a sugestão de novos testes psicométricos da versão para estudantes, sem o referido item, em diferentes contextos.

O INE-R *Survey* - versão brasileira permite apoiar estudos e estratégias que necessitam medidas de percepção ou de frequência de comportamentos incivis no ambiente de ensino superior de enfermagem.

# Translation: **Propiedades psicométricas del *Incivility in Nursing Education - Revised Survey* versión brasileña** *

**DOI:** 10.1590/1518-8345.6950.4214

**Authors:** Vanessa dos Santos Ribeiro, Cynthia M. Clark, Claudia Benedita dos Santos, João Marôco, Jonas Bodini Alonso, Aline Helena Appoloni Eduardo, Emilia Campos de Carvalho

**Affiliations:** 1 Universidade de São Paulo, Escola de Enfermagem de Ribeirão Preto, Centro Colaborador de la OPS/OMS para el Desarrollo de la Investigación en Enfermería, Ribeirão Preto, SP, Brasil.; 2 Becaria de la Coordenação de Aperfeiçoamento de Pessoal de Nível Superior (CAPES), Brasil.; 3 Boise State University, School of Nursing, Boise, Idaho, ID, Estados Unidos de América.; 4 Becaria del Conselho Nacional de Desenvolvimento Científico e Tecnológico (CNPq), Brasil.; 5 William James Center for Research, ISPA - Instituto Universitário, Lisboa, Portugal.; 6 Universidade Federal de São Carlos, Departamento de Enfermagem, São Carlos, SP, Brasil.

**Keywords:** Enfermería, Incivilidad, Estudios de Validación, Educación, Estudiantes de Enfermería, Maestros

## Abstract

**Objetivo:**

analizar las propiedades psicométricas de la versión brasileña del *Incivility in Nursing Education* - *Revised Survey* con estudiantes de graduación en enfermería.

**Método:**

estudio metodológico, realizado en una escuela de enfermería del estado de São Paulo. Se trata del análisis de las propiedades psicométricas (confiabilidad y validez de constructo) de los ítems del INE-R *Survey* - versión brasileña. La validez de constructo fue realizada por Análisis Factorial Confirmatorio y la confiabilidad por el test-retest, para verificación de la estabilidad del instrumento, calculada por el Coeficiente de Correlación Intraclase y por medio de la consistencia interna de los ítems, según los coeficientes alfa de Cronbach, alfa ordinal y omega de McDonald*.*

**Resultados:**

hubo ajuste del modelo propuesto del análisis factorial confirmatorio con dos factores (baja y alta incivilidad), con sugerencia de exclusión de un ítem para estudiantes. La mayoría de los valores de ajustes, para los ítems de los estudiantes y la totalidad para los profesores, atendió las referencias establecidas por la literatura; los valores para los Coeficientes de Consistencia Interna fueron mayores que 0,80 y los Coeficientes de Correlación Intraclase mayores que 0,75.

**Conclusión:**

la versión brasileña del *Incivility in Nursing Education* - *Revised Survey* se encuentra validada para el contexto estudiado, presentando confiabilidad satisfactoria y validez, por el análisis factorial que confirmó el modelo original con dos factores, con 23 ítems, retratando comportamientos de estudiantes y 24 de profesores.

## Destacados

**(1)** La incivilidad es un fenómeno global y creciente en la enseñanza superior.

**(2)** El ambiente incivil interfiere en el aprendizaje y en los indicadores de salud.

**(3)** Presentamos una *survey* validada para medir la incivilidad en la enseñanza de enfermería.

## Introducción

Por más de cuatro décadas, el ambiente de la enseñanza superior, incluyendo el de Enfermería, ha sido escenario de comportamiento incivil, es decir, comportamiento que no atiende al cumplimiento de reglas sociales para la convivencia social^1^ *.* Incivilidad es cualquier comportamiento que estorbe el proceso de aprendizaje e interfiera en el ambiente de aprendizaje cooperativo. 

Algunas veces descrita como abuso de poder sobre otros^2^, la incivilidad puede ser expresada como comportamientos dirigidos a otra persona, desde formas menos agresivas como insultos, desacuerdos y conflictos o, por formas más serias de comportamientos agresivos, tal como la violencia física entre individuos. La incivilidad puede también representar cualquier diálogo, interacción o actitud que afecta desfavorablemente el bienestar de estudiantes o docentes, debilitando las relaciones profesionales, bloqueando el proceso de enseñanza-aprendizaje e interfiriendo en la calidad de la salud física y mental^3^.

Si permitidos los comportamientos inciviles pueden suscitar un proceso en cadena, en el cual se genera cada vez más incivilidades. En ese sentido, profesores, tutores y gestores académicos deben estar aptos para reducir esos comportamientos, en los distintos ambientes educacionales, evitando que incivilidades impacten negativamente en las relaciones y aprendizaje, tanto teórico como práctico, comprometiendo la formación del futuro enfermero^4^ ^)-(^ ^5^.

La presencia de incivilidad, tanto en el ambiente de enseñanza como en la práctica de enfermería, es un fenómeno global^6^, constatado por estudios realizados en distintas culturas, como en Irán^7^ ^)-(^ ^8^, Emiratos Árabes^6^, Afganistán^9^, Canadá^(10^, Italia^11^, China^12^, Indonesia^13^, Corea^14^, Turquía^15^ y Estados Unidos de América^16^ ^)-(^ ^17^, entre otros. A pesar de ser global, un estudio reciente multicéntrico, con docentes de enfermería de 10 países, identificó que este es un fenómeno percibido de manera diferente entre los países que participaron del estudio^18^. 

Los actos inciviles u otras formas de agresión ocurren tanto en las relaciones presenciales como en las relaciones virtuales, que frecuentemente tratan de las comunicaciones en redes sociales o en actividades académicas remotas; en la enfermería se observó el crecimiento de comportamientos inciviles en la enseñanza *online* ^19^. Se trata de un proceso complejo y multidireccional, en que estudiantes, profesores y preceptores pueden contribuir para un clima de falta de respeto, de uno con el otro, o durante el proceso de aprendizaje, empleando comportamientos relacionados al género^20^, a no comparecer a las clases^21^, a respuestas desnecesarias^22^, e actos impulsivos y agresivos exhibidos *online* ^23^, entre otros. 

Estudios apuntan la correlación positiva entre estrés y comportamientos de incivilidad^4^ ^),(^ ^23^ ^)-(^ ^24^. Durante la pandemia de COVID-19, un período en que las clases remotas fueron potencializadas, algunas investigaciones informaron un aumento del estrés, ansiedad y depresión, referido por estudiantes de enfermería^16^.

Como fue destacado en el modelo conceptual de incivilidad en educación de enfermería^25^, a partir de la percepción de la presencia de comportamientos inciviles, sobre todo de profesores, los estudiantes responden con: posibilidad de mantenerse en la institución y conformarse de acuerdo a lo esperado (*loyalty*); de desafiar el *status quo* y esforzarse para cambiar esos comportamientos en la institución (*voice*); o, de dejar la escuela (*exit*). Las respuestas emocionales, expectativas y decisiones son dependientes del soporte que el estudiante pueda recibir, lo que contribuirá para el éxito de la conclusión del curso^25^. 

Los rompimientos de las normas acordadas para el ambiente escolar, presencial o remoto, por los estudiantes o profesores, perjudican el proceso de enseñanza-aprendizaje; estos deben ser identificados, así como sus factores causales, para que puedan ser administradas y enfrentadas.

Uno de los instrumentos creados para medir comportamientos no civilizado es el *Incivility in Nursing Education Survey* (INE). Al construir el INE y en sus sucesivas revisiones, la autora buscó identificar las percepciones de estudiantes y profesionales sobre los comportamientos que representarían la amplitud del fenómeno de incivilidad. Estudios desarrollados en distintas culturas o con profesionales y estudiantes, de diferentes tipos de programas de enfermería, apuntan similitudes en esos comportamientos. Evidencias usadas para desarrollar y revisar el INE-R^3^ describen la base para inclusión de comportamientos específicos de estudiantes y profesores. Esos comportamientos retratan un *continuum*, denominado *Continuum of Workplace Aggresion* ^26^, que caracteriza una estructura confiable con una serie de comportamientos inciviles, la cual comprende desde comportamientos disruptivos o de bajo nivel de incivilidad, hasta comportamientos más serios como violencia física o tragedias; estos comportamientos cuando relacionados a los estudiantes, contemplan distracciones o falta de respeto en la sala de clases, falta de respeto por el otro o, también, desinterés general por la clase^27^. 

El INE-R, desarrollado en 2004, está compuesto por: definición de incivilidad; por datos de identificación del encuestado, por un conjunto de ítems en que constan los comportamientos inciviles practicados por profesores; otro conjunto de ítems con comportamientos inciviles practicados por los estudiantes; y, además de preguntas evaluativas. El proceso de elaboración del INE y de sus revisiones está descrito en la literatura^28^ ^)-(^ ^29^. En 2014 el INE-R original fue revisado y renombrado como *Incivility in Nursing Education - Revised (INE-R) Survey*, en el cual algunos ítems fueron revisados y se añadieron dos preguntas con espacios en blanco, para registro discursivo sobre las experiencias de los encuestados con incivilidad académica y modos de prevenir y enfrentar el problema. La versión INE-R fue validada entre estudiantes y profesores de enfermería norteamericanos y obtuvo resultados adecuados de su estructura y confiabilidad^3^ ^),(^ ^7^. 

El INE-R^3^ fue también validado con estudiantes coreanos^14^ y árabes^30^. Delante de los éxitos de las investigaciones que lo emplearon y de la importancia de contar con un instrumento válido y confiable, para medir comportamientos inciviles, figuró como fue objeto del presente estudio, cuyo objetivo fue analizar las propiedades psicométricas de la versión brasileña del *Incivility in Nursing Education - Revised Survey* con estudiantes de graduación en enfermería.

## Método

### Tipo de estudio, local y período

Se trata de un estudio metodológico referente a las etapas de verificación de la validez, según análisis de la estructura factorial y de la confiabilidad, realizado en una institución pública de enseñanza superior en enfermería de una ciudad del interior del estado de Sao Paulo, Brasil, de junio de 2021 a marzo de 2022. Preliminarmente, a la etapa del estudio descrita, se procedió a la adaptación cultural y evaluación semántica del INE-R^31^. 

### Participantes y criterios de selección

Fueron invitados a participar del estudio todos los estudiantes de graduación (n = 440) en enfermería, regularmente matriculados en la institución, excepto los del primer año lectivo, debido a que los participantes deberían tener previamente, por lo menos 12 meses de vivencia en el curso, para responder al INE-R^3^. De los elegibles - es decir, todos estudiantes del curso de graduación excepto los del primer año - 60% aceptaron participar del estudio. No hubo solicitación de abandono durante el llenado del instrumento.

### Recolección de datos e instrumentos utilizados

El reclutamiento y la recolección de datos fueron realizados después de la aquiescencia de la institución y del colegiado responsable por la enseñanza de graduación. La invitación y el suministro del consentimiento informado y de los instrumentos de recolección fueron elaborados por una de las autoras y suministrados cada estudiante, durante los intervalos de las actividades académicas. En esas ocasiones, fueron informados que podrían ser sorteados para la fase de postest, prevista para ocurrir aproximadamente dentro de 15 días, conforme recomendaciones de la literatura^32^.

A continuación, cada estudiante llenó el Término de Consentimiento Libre e Informado; a seguir respondió al INE-R *Survey* versión brasileña, individualmente, en la presencia del investigador. Para el análisis de la estabilidad del instrumento, 60 estudiantes fueron sorteados para responder nuevamente a la *Survey*. 

El original INE-R *Survey* ^3^ ^)^ es autoaplicable, organizado en secciones, iniciándose por la conceptualización de incivilidad y de ambiente académico, seguido de los datos demográficos del encuestado (estudiante o profesor), que serán determinados a criterio del estudio en desarrollo, tales como, género, edad, origen étnica/racial, años de formación, programa al cual el encuestado pertenece, posición/cargo del docente o semestre lectivo del estudiante, entre otros. En este estudio fueron considerados la edad, las identidades de género y étnico-raciales y el semestre lectivo del encuestado.

La sección siguiente presenta una lista de 48 ítems divididos en dos grupos, siendo uno con 24 ítems relativos a los comportamientos de estudiantes y el otro con 24 ítems relacionados al comportamiento de profesores. Ambos presentan dos factores - Alta Incivilidad y baja incivilidad - compuestos por nueve y quince ítems, respectivamente. Cada ítem es evaluado por una escala Likert que corresponde al nivel de incivilidad que el comportamiento representa al encuestado, con alternativas entre 1 a 4, las que fueron: no incivil; poco incivil, moderadamente incivil y altamente incivil. Cada ítem también es evaluado en cuanto a la frecuencia del comportamiento en los últimos 12 meses, con cuatro alternativas de respuestas (escala de 1 a 4) correspondiendo respectivamente a: nunca; raramente; a veces y frecuentemente. Dos preguntas completan la segunda sesión, abordando cómo el encuestado percebe la magnitud del fenómeno en la institución (evaluada entre cuatro alternativas: desde no es problema hasta es problema muy serio; y, como cada encuestado percebe la probabilidad de participación de profesores y estudiantes en actos inciviles evaluada con las alternativas: mucho mayor del profesor, mayor del profesor, ambos, mayor del alumno y mucho mayor de alumnos^3^. 

La última sección presenta un conjunto de preguntas y un espacio narrativo, para que el encuestado describa situaciones, opine sobre: incivilidad en la educación en enfermería; comportamientos inciviles de estudiantes y profesores; nivel de civilidad en la institución; priorización de estrategias para elevar el nivel de civilidad; y, causas y consecuencias de la incivilidad académica^3^.

### Tratamiento y análisis de los datos

Los datos de las diferentes variables fueron introducidos, en doble digitación, en una planilla electrónica del aplicativo Excel, constituyendo un banco de datos estructurado. Para las variables semestre lectivo, etnia y género, se utilizó la estadística descriptiva; para la variable edad, como medida de tendencia central, fueron establecidas la media y la mediana; y, como medida de dispersión, la desviación estándar.

Fueron realizados los análisis de las propiedades psicométricas (confiabilidad y validez de constructo) de los ítems del INE-R *Survey* - versión brasileña. Preliminarmente, la normalidad de los datos fue verificada por los valores absolutos de asimetría (Sk) y de curtosis (Ku)^33^. La validez de constructo fue realizada con el Análisis Factorial Confirmatorio y la confiabilidad con el test-retest; para verificación de la estabilidad del instrumento, calculada por el Coeficiente de Correlación Intraclase (ICC) y por medio de la consistencia interna de los ítems, según coeficientes alfa de Cronbach, ordinal y omega de McDonald^32^. La razón del número de participantes fue calculada por el número de ítems del instrumento, lo que limitó otros análisis.

El Análisis Factorial Confirmatorio fue realizado con el *software* R (R *Core Team*, 2021) versión 3.4.2 y con el uso del paquete *Latent Variable Analisis* (LAVAAN)^34^. Para estimación de los parámetros fue utilizado el método de mínimos cuadrados ponderados diagonalmente (*Diagonally Weighted Least Squares* - DWLS)^35^.

En relación al análisis de la calidad del ajuste del modelo, los índices de ajuste absoluto, índices de ajuste incremental e índices de discrepancia poblacional, fueron tomados como alternativas para la verificación del ajuste^35^. El índice de discrepancia poblacional investigado fue la Raíz del Error Cuadrático Medio de Aproximación (*Root Mean Square Error of Aproximation* - RMSEA), que estima si los parámetros del modelo reproducen la covariancia poblacional en esas condiciones, el RMSEA tiende a ser igual a cero. También se evaluó la Raíz del Error Cuadrático Medio Residual (*Standardized root mean square residual* - SRMR), o sea, la diferencia estandarizada entre la correlación observada y la prevista. Valores inferiores a 0,10 indican ajustamiento aceptable para RMSEA e inferiores a 0,08 son considerados aceptables para SRMR^35^. 

En relación a los índices de ajustes incrementales, fueron utilizados el Índice de Ajuste Normalizado (*Normed Fit Index* - NFI), el Índice de Ajuste Comparativo (*Comparative Fit Index* - CFI) y el Índice de Tucker-Lewis (TLI). Los valores de NFI y CFI, así como el TLI, pueden encontrarse entre cero y uno; los valores arriba de 0,90 indican un ajuste aceptable^35^.

Para analizar la confiabilidad se utilizó la consistencia interna determinada por el coeficiente alfa (α) de Cronbach. Este indicador permite comparar los resultados obtenidos con los demás estudios que validaron el instrumento. Además, también fueron analizados los valores de los coeficientes alfa ordinal y omega de McDonald (ω). Para cada dominio del instrumento (alto y bajo nivel de incivilidad), en cada grupo de ítems (los relativos a comportamientos de estudiantes y de profesores). Los valores de α de Cronbach, de alfa ordinal y omega de McDonald variaron entre cero y uno; en este estudio, los valores iguales o mayores que 0,70 fueron considerados aceptables^32^. 

Para verificación de la estabilidad de la medida, se realizó el test-retest con obtención del Coeficiente de Correlación Intraclase (ICC), de forma semejante a los demás estudios que validaron ese instrumento. En este análisis, fue considerado el nivel de significación de 5% (α= 0,05). Los valores del ICC entre 0,70 y 0,90 son considerados buenos y mayores que 0,90 son considerados excelentes^32^.

### Aspectos éticos

Los participantes fueron invitados, asegurándoles la total privacidad de sus informaciones. La investigación solamente fue iniciada después de la aprobación de la Comisión de Enseñanza y del Comité de Ética en Investigación con Seres Humanos (CEI), ambas de la institución en la cual el estudio fue realizado (Dictamen CEP: 3.635.814 y Certificado de Presentación de Apreciación Ética-CAAE 18508919.4.0000.5393).

## Resultados

Participaron del estudio 264 estudiantes. En relación a las identidades de género y étnico-raciales 222 (84,1%) se identificaron como mujeres; 40 (15,2%) como hombres, uno (0,4%) como ambos y otro (0,4%) como ninguno; 180 (68,2%) se declararon blancos, 57 (21,6%) pardos, 22 (8,3%) negros, 4 (1,5%) amarillos, en cuanto 1 (0,4%) se denominó indígena. Entre los estudiantes, 177 (67,0%) estaban en los años intermediarios (tercer al sexto semestre lectivo) y 87 (33,0%) estaban en los años de conclusión (séptimo semestre en adelante).

Los valores medios respectivos - de desviación estándar, asimetría (Sk) y curtosis (Ku) de los niveles de incivilidad de comportamientos, cuando emitidos por estudiantes y por profesores, según los participantes (n = 264) - se presentan en la Tabla 1. Al considerarse los valores referentes a la asimetría y curtosis de cada ítem, estos fueron inferiores a tres y siete, respectivamente, para todos los ítems, comprobando la no violación de la normalidad, presupuesto para realización de la AFC.

**Tabla 1:** Estadística descriptiva (medias, desviación estándar, asimetría y curtosis) de los niveles de incivilidad de comportamientos cuando emitidos por estudiantes y por profesores, según los participantes (n = 264). Interior del estado de São Paulo, Brasil, 2021-2022

Comportamientos	Ítem	Media	Desviación estándar	Asimetría	Curtosis
**Relacionados a los estudiantes**	01	2,76	0,84	-0,33	-0,45
	02	3,17	1,11	-0,94	-0,65
	03	2,87	0,82	-0,37	-0,37
	04	2,21	0,93	0,40	-0,70
	05	3,12	0,82	-0,77	0,15
	06	2,64	0,86	-0,27	-0,57
	07	2,48	0,84	-0,08	-0,60
	08	2,58	0,85	-0,01	-0,65
	09	2,49	0,92	0,10	-0,85
	10	3,08	1,01	-0,73	-0,70
	11	3,02	1,08	-0,72	-0,85
	12	3,17	0,85	-0,81	-0,01
	13	3,31	0,93	-1,13	0,12
	14	3,43	1,10	-1,56	0,61
	15	2,21	1,00	0,40	-0,90
	16	3,14	1,10	-0,94	-0,57
	17	3,05	1,12	-0,77	-0,88
	18	2,54	0,95	-0,10	-0,93
	19	3,42	1,11	-1,56	0,63
	20	3,51	1,08	-1,80	1,33
	21	3,44	1,04	-1,58	0,87
	22	3,47	1,12	-1,69	0,91
	23	3,45	1,13	-1,63	0,75
	24	3,47	1,14	-1,69	0,88
Relacionados a los profesores	01	3,28	0,86	-1,08	0,04
	02	3,49	0,95	-1,05	-0,08
	03	3,31	0,86	-1,48	1,42
	04	3,09	0,92	-1,84	1,91
	05	3,01	0,96	-0,73	-0,33
	06	2,87	0,85	-2,23	4,02
	07	2,60	0,94	-2,40	4,48
	08	3,26	0,96	-1,56	1,87
	09	3,22	0,99	-1,49	0,97
	10	3,49	0,78	-2,74	6,71
	11	3,53	0,95	-0,91	0,16
	12	3,01	0,94	-1,82	1,49
	13	3,64	0,78	-2,01	2,21
	14	3,66	0,82	-1,87	1,73
	15	3,46	0,81	-1,88	1,58
	16	3,40	0,96	-1,86	1,56
	17	3,73	0,71	-1,88	1,60
	18	3,21	0,85	-1,08	0,04
	19	3,51	1,05	-1,05	-0,08
	20	3,57	1,01	-1,48	1,42
	21	3,53	1,02	-1,84	1,91
	22	3,53	1,08	-0,73	-0,33
	23	3,52	1,07	-2,23	4,02
	24	3,53	1,07	-2,40	4,48

Al considerar los dominios, las medias del nivel de incivilidad de los comportamientos de estudiantes fueron: Dominio Baja Incivilidad x = 2,76 y de x = 0,53; Dominio Alta Incivilidad x = 3,39 y de x = 0,97; y para los profesores fueron: Dominio Baja Incivilidad x = 3,22 y de x =0,69; Dominio Alta Incivilidad x = 3,58 y de x = 0,89. La mediana de los ítems de incivilidad atribuidos a los estudiantes para el Dominio Baja Incivilidad fue 3 y para el Dominio Alta Incivilidad fue 4; la atribuida a los comportamientos de docentes para el Dominio Baja Incivilidad fue 3; y para el Dominio Alta Incivilidad fue 4.

En relación a la frecuencia de comportamientos inciviles en los últimos 12 meses**,** las medias y respectivas desviaciones estándar para los ítems relativos a comportamientos de docentes fueron: Dominio Baja Incivilidad x = 2,04 y de x = 0,49; Dominio Alta Incivilidad x = 1,46 y de x = 0,35; y para los relativos a los de los estudiantes fueron Dominio Baja Incivilidad x = 2,43 y de x = 0,53; y, Dominio Alta Incivilidad x =1,54 y de x = 0,49. Las medianas, valores mínimos y máximos para todos los ítems fueron iguales para estudiante y profesores (mediana = 2; mínimo = 1; máximo = 4), baja y alta incivilidad.

En la AFC, entre los comportamientos de estudiantes, todos los ítems del Dominio Alta Incivilidad presentaron cargas factoriales elevadas, variando de 0,734 a 0,999. Para el Dominio de Baja Incivilidad, tres ítems (3, 4 y 8) presentaron cargas variando de 0,304 a 0,396 y los demás presentaron valores variando de 0,613 a 0,825, excepto los ítems 1, 5 y 15 que presentaron carga factorial < 0,300. El ítem 1) expresar desinterés, aburrimiento o apatía sobre el contenido del curso o asunto, presentó carga factorial 0,149, IC[95%]: [0,105; 0,192]; el ítem 5) usar computador, celular u otros dispositivos electrónicos durante la clase, reunión o actividad para fines no relacionados - presentó carga factorial igual a 0,290, IC[95%]: [0,249; 0,332] ; y el ítem 15) exigir pruebas substitutas, extensiones de plazos u otros favores especiales - presentó carga factorial de 0,299; IC[95%]: [0,259; 0,338] (Tabla 2).

**Tabla 2:** Resultados del Análisis Factorial Confirmatorio en relación a las cargas factoriales de los ítems referentes a los comportamientos de estudiantes (n = 264). Interior del estado de São Paulo, Brasil, 2021-2022

Dominio	Ítem	Cargas factoriales	Intervalo de confianza 95%	Error-estándar	Z*	***P* valor**
**Alta Incivilidad**	Ítem13	0,734	(0,702; 0,766)	0,039	18,916	0,000
	Ítem 14	0,946	(0,930; 0,962)	0,016	57,785	0,000
	Ítem17	0,855	(0,833; 0,877)	0,023	37,411	0,000
	Ítem 19	0,988	(0,983; 0,993)	0,004	236,035	0,000
	Ítem 20	0,999	(0,997; 1,000)	0,001	1431,998	0,000
	Ítem 21	0,958	(0,947; 0,969)	0,011	85,421	0,000
	Ítem 22	0,999	(0,997; 1,000)	0,001	941,880	0,000
	Ítem 23	0,999	(0,997; 1,000)	0,001	1210,095	0,000
	Ítem 24	0,999	(0,997; 1,000)	0,001	740,119	0,000
**Baja Incivilidad**	Ítem 1	0,149	(0,105; 0,192)	0,070	2,119	0,000
	Ítem 2	0,802	(0,772; 0,831)	0,032	24,995	0,000
	Ítem 3	0,350	(0,311; 0,388)	0,060	5,861	0,000
	Ítem 4	0,304	(0,261; 0,346)	0,066	4,618	0,000
	Ítem 5	0,290	(0,249; 0,332)	0,062	4,653	0,000
	Ítem 6	0,641	(0,604; 0,678)	0,043	15,021	0,000
	Ítem 7	0,538	(0,500; 0,576)	0,048	11,188	0,000
	Ítem 8	0,396	(0,354; 0,438)	0,060	6,643	0,000
	Ítem 9	0,577	(0,537; 0,616)	0,050	11,587	0,000
	Ítem 10	0,825	(0,798; 0,853)	0,028	29,948	0,000
	Ítem 11	0,825	(0,798; 0,852)	0,030	27,392	0,000
	Ítem 12	0,758	(0,728; 0,788)	0,034	22,184	0,000
	Ítem 15	0,299	(0,259; 0,338)	0,062	4,801	0,000
	Ítem 16	0,776	(0,746; 0,805)	0,036	21,609	0,000
	Ítem 18	0,613	(0,581; 0,644)	0,041	14,809	0,000

*Z = Variable normal estandarizada

En lo que se refiere a los comportamientos de profesores, la menor de las cargas factoriales de los ítems fue 0,596 (ítem 18), la que pertenece al Dominio Baja Incivilidad, y las mayores (ítems 20, 22, 23 y 24) pertenecen al Dominio Alta Incivilidad; por tanto, todos los ítems tuvieron cargas factoriales satisfactorias (Tabla 3).

**Tabla 3:** Resultados del Análisis Factorial Confirmatorio (AFC) en relación a las cargas factoriales de los ítems relativos a comportamientos de profesores (n = 264). Interior del estado de São Paulo, Brasil, 2021-2022

Dominio	Ítem	Cargas factoriales	Intervalo de confianza 95%	Error-estándar	Z*	***P* valor**
**Alta Incivilidad**	Ítem13	0,888	(0,870; 0,906)	0,026	33,940	0,000
	Ítem 14	0,963	(0,952; 0,975)	0,013	73,082	0,000
	Ítem17	0,948	(0,935; 0,961)	0,016	59,428	0,000
	Ítem 19	0,990	(0,985; 0,995)	0,004	241,701	0,000
	Ítem 20	0,997	(0,995; 0,999)	0,002	606,938	0,000
	Ítem 21	0,993	(0,989; 0,996)	0,003	313,800	0,000
	Ítem 22	1,000	(1,000; 1,000)	0,001	1203,589	0,000
	Ítem 23	0,998	(0,995; 1,000)	0,001	729,320	0,000
	Ítem 24	0,998	(0,996; 1,000)	0,001	739,529	0,000
**Baja Incivilidad**	Ítem 1	0,827	(0,807; 0,848)	0,026	31,722	0,000
	Ítem 2	0,930	(0,914; 0,946)	0,020	47,166	0,000
	Ítem 3	0,674	(0,647; 0,701)	0,043	15,696	0,000
	Ítem 4	0,816	(0,706; 0,836)	0,029	28,355	0,000
	Ítem 5	0,807	(0,786; 0,827)	0,024	33,571	0,000
	Ítem 6	0,762	(0,739; 0,785)	0,028	26,902	0,000
	Ítem 7	0,709	(0,684; 0,734)	0,036	19,748	0,000
	Ítem 8	0,899	(0,882; 0,917)	0,018	51,310	0,000
	Ítem 9	0,891	(0,873; 0,909)	0,020	44,766	0,000
	Ítem 10	0,779	(0,756; 0,803)	0,033	23,809	0,000
	Ítem 11	0,937	(0,921; 0,954)	0,017	54,505	0,000
	Ítem 12	0,814	(0,794; 0,834)	0,026	31,101	0,000
	Ítem 15	0,782	(0,758; 0,805)	0,034	23,185	0,000
	Ítem 16	0,900	(0,883; 0,917)	0,020	45,231	0,000
	Ítem 18	0,596	(0,567; 0,625)	0,050	12,002	0,000

*Z = Variable normal estandarizada

A partir de las estimativas puntuales y por los intervalos de las cargas factoriales de los ítems, de la versión brasileña del instrumento, se sugiere una organización estructural de los ítems con dos factores, Alta Incivilidad y baja incivilidad, semejante a la estructura original, con la exclusión del ítem 1 para los estudiantes, que presentó IC95% para la carga factorial incluyendo solamente valores inferiores a 0,30.

Los resultados del Análisis Factorial Confirmatorio en relación a las cargas factoriales de los ítems relativos a comportamientos de estudiantes, con la exclusión del ítem 1 para estudiantes, se presentan en la Tabla 4.

**Tabla 4:** Resultados del Análisis Factorial Confirmatorio en relación a las cargas factoriales de los ítems relativos a comportamientos de estudiantes (n = 264). Interior del estado de São Paulo, Brasil, 2021-2022

Dominio	Ítem	Cargas factoriales	Intervalo e confianza 95%	Error-estándar	Z*	***P* valor**
**Alta Incivilidad**	Ítem13	0,734	(0,702; 0,766)	0,039	18,865	0,000
	Ítem 14	0,946	(0,930; 0,962)	0,016	57,844	0,000
	Ítem17	0,855	(0,834; 0,877)	0,023	37,411	0,000
	Ítem 19	0,988	(0,983; 0,993)	0,004	236,087	0,000
	Ítem 20	0,999	(0,997; 1,000)	0,001	1431,743	0,000
	Ítem 21	0,958	(0,947; 0,969)	0,011	85,376	0,000
	Ítem 22	0,999	(0,997; 1,000)	0,001	941,885	0,000
	Ítem 23	0,999	(0,997; 1,000)	0,001	1210,134	0,000
	Ítem 24	0,999	(0,997; 1,000)	0,001	740,191	0,000
**Baja Incivilidad**	Ítem 2	0,801	(0,771; 0,830)	0,032	24,968	0,000
	Ítem 3	0,346	(0,307; 0,384)	0,060	5,768	0,000
	Ítem 4	0,301	(0,258; 0,343)	0,066	4,565	0,000
	Ítem 5	0,287	(0,246; 0,328)	0,063	4,579	0,000
	Ítem 6	0,640	(0,604; 0,677)	0,043	14,967	0,000
	Ítem 7	0,535	(0,497; 0,572)	0,048	11,058	0,000
	Ítem 8	0,394	(0,352; 0,436)	0,060	6,579	0,000
	Ítem 9	0,575	(0,536; 0,615)	0,050	11,535	0,000
	Ítem 10	0,825	(0,798; 0,852)	0,028	29,942	0,000
	Ítem 11	0,824	(0,797; 0,852)	0,030	27,339	0,000
	Ítem 12	0,757	(0,728; 0,787)	0,034	22,143	0,000
	Ítem 15	0,299	(0,259; 0,338)	0,062	4,794	0,000
	Ítem 16	0,775	(0,746; 0,805)	0,036	21,579	0,000
	Ítem 18	0,612	(0,580; 0,644)	0,041	14,791	0,000

*Z = Variable normal estandarizada

Delante de los resultados presentados en la Tabla 4, con la exclusión del ítem 1, y por las justificativas anteriormente presentadas, la opción fue por la manutención de todos los demás 23 ítems para los estudiantes.

Para la evaluación de constructo, a partir del Análisis Factorial Confirmatorio, las medidas adoptadas de ajuste del modelo a la población, comprobadas para los ítems que retratan comportamientos de estudiantes y profesores, respectivamente, fueron: Xi cuadrado (χ^2^ = 815,609 y 491,440); Grados de Libertad (GL= 229; 251); (χ^2^/GL = 3,56 y 1,96); *p*-valores (< 0,001; < 0,001); Índice de Ajuste Comparativo (CFI = 1,000; 1,000); Índice de Tucker-Lewis o Indicie de Ajuste No Normalizado (TLI = 1,000; 1,000); Índice de Ajuste Normalizado (NFI =1,000; 1,000); Raíz de Error Cuadrático Medio de Aproximación (RMSEA = 0,092; 0,044); Intervalo con 95% de Confianza para RMSEA (0,083; 0,101) y (0,035; 0,055) y Raíz del Error Cuadrático Medio Residual (SRMR = 0,107; 0,053). La mayoría de los índices para estudiantes y todos los índices para los profesores indican un ajustamiento aceptable.

Las representaciones de los Dominios Baja incivilidad y Alta Incivilidad y de sus ítems del INE-R-versión brasileña, con sus respectivas cargas factoriales, del Análisis Factorial Confirmatorio, se presentan en la Figura 1. La estructura presentada para profesores es conforme a lo propuesto en el modelo original del instrumento, y para estudiantes, respetando las justificativas constantes en la discusión sobre los ítems.

**Figura 1:** Diagrama de flujo de los caminos de los Dominios Baja Incivilidad y Alta Incivilidad del *Incivility in Nursing Education - Revised Survey* versión brasileña, considerando la estructura factorial original. Interior del estado de Sao Paulo, Brasil, 2021-2022 *It =ítems; ^†^BxInv = Bajo nivel de incivilidad; ^‡^AltInv = Alto nivel de incivilidad

La confiabilidad del INE-R *Survey* - versión brasileña fue evaluada por la consistencia interna y por la reproductibilidad. La consistencia interna evaluada por los coeficientes alfa de Cronbach, alfa ordinal y omega de McDonald, se presenta en la Tabla 5.

**Tabla 5:** Consistencia interna del *Incivility in Nursing Education - Revised Survey* versión brasileña, según el grupo de comportamientos inciviles, las dimensiones de incivilidad e ítems que los componen (n = 264). Interior del estado de Sao Paulo, Brasil, 2021-2022

Grupo de Comportamientos	Dimensiones de incivilidad	Ítems	Valor de Alfa Cronbach (IC 95%)	Valor Alfa ordinal	Valor de Omega
**Estudiante**	Alta	13, 14, 17, 19, 20, 21, 22, 23, 24	0,966 (0,962; 0,974)	0,984	0,976
	Baja	2 a 12, 15, 16, 18	0,853 (0,827; 0,879)	0,880	0,857
**Profesor**	Alta	13, 14, 17, 19, 20, 21, 22, 23, 24	0,977 (0,973; 0,981)	0,993	0,989
	Baja	1 a 12, 15, 16, 18	0,949 (0,940; 0,958)	0,964	0,951

La consistencia interna puede ser considerada altamente satisfactoria, tanto para los ítems que evalúan los comportamientos de los estudiantes y de profesores, como para ambos dominios de incivilidad, evidenciando adecuada confiabilidad en la muestra estudiada. 

En lo que se refiere al análisis de la reproductibilidad del instrumento, de los 60 estudiantes sorteados para esta fase, solamente 29 participaron. En relación al nivel de incivilidad, la estabilidad, analizada por el Coeficiente de Correlación Intraclase, fue considerada con concordancia buena con valor de ICC, en relación a los ítems para los comportamientos de estudiantes (ICC = 0,612, IC[95%]: [0,330; 0,796] y *p*-valor < 0,0001) y moderada para los comportamientos de los profesores (ICC = 0,598 IC[95%]:[0,301; 0,789] y *p*-valor < 0,0001).

Para la estabilidad de los ítems que retratan la frecuencia de los comportamientos inciviles observados en los últimos 12 meses, en las fases de pretest y postest, la concordancia fue considerada moderada para los estudiantes (0,474, IC [95%]: [0,148; 0,710] y *p*-valor = 0,003) y, buena para los profesores (ICC = 0,615, IC [95%]: [0,332; 0,789] y *p*-valor < 0,0001). 

## Discusión

En este estudio se analizaron las propiedades psicométricas de la versión brasileña del INE-R *Survey* ^3^. El INE_R *survey* fue útil para identificar las percepciones de actos inciviles de alumnos y de profesores de enfermería^18^, permitiendo establecer las distinciones de las percepciones de los dos grupos^7^. 

En relación al instrumento, la variable del estudio es latente y fue medida por medio de opciones de respuestas, en escala del tipo Likert, que varían de 1 a 4, por tanto no es simétrica (no existe punto neutro equidistante de los extremos) y no es homogénea en relación al número de ítems, ya que cada dominio tiene, respectivamente, nueve y 15 ítems^36^. 

El período de la recolección de datos se realizó durante la vigencia de la pandemia de la COVID-19, ocasión en la cual las actividades de estudiantes y docentes se presentaban en fase de ajustes, con la introducción de enseñanza *online.* Esta situación provocó sobrecargas de demandas y dificultades de acceso y manoseo de dispositivos^37^. Según la literatura, cambios en las percepciones de frecuencia o nivel de incivilidades de comportamientos de estudiantes y profesores fueron identificadas en el período de la pandemia^38^ ^)-(^ ^39^.

En este estudio los ítems correspondientes a los comportamientos de estudiantes y de profesores fueron evaluados apenas por estudiantes. El estudio original norteamericano validó el instrumento por profesores y estudiantes, y también para los conjuntos de ítems de comportamientos de estudiantes y de profesores^3^. Los otros estudios validaron el instrumento entero apenas con estudiantes^14^ ^)^ o solamente los ítems relativos a los comportamientos de estudiantes^30^. El instrumento puede ser evaluado para cada uno de los sujetos objeto del estudio (estudiantes o profesores) o conjuntamente^3^.

Las percepciones referidas sobre comportamientos de profesores, en este estudio, se refieren a las experiencias con docentes de una institución pública, secular (no religiosa), que en su mayoría trabaja por largos períodos en la misma institución. La literatura cita un estudio realizado en África del Sur^40^, en el cual el tiempo de trabajo en la misma institución presentó asociación con el aumento de la incivilidad; otro estudio menciona haber niveles aumentados de incivilidad en la academia^41^. Estos aspectos pueden haber generado impacto en la expresión de comportamientos inciviles.

Por tanto, el tipo prolongado de vínculo del profesor en la institución^40^, el hecho de la recolección de datos haber ocurrido durante un período de ajustes a nuevas demandas impuestas por la pandemia^16^ ^)^ y el aumento del uso de tecnologías remotas para las clases^38^ ^),(^ ^42^, durante o en los semestres anteriores a la realización del estudio, pudieron haber impactado las respuestas del estudiante, es decir, en la percepción de frecuencia o del nivel de comportamientos de incivilidades.

En relación al número de participantes, al emplearse un instrumento como el descrito, existe indicación en la literatura que para realizar el análisis factorial, sean computados de 5 a 10 encuestados por cada pregunta del instrumento. Sin embargo, al utilizar un modelo de ecuaciones estructurales, la literatura que recomienda de 10 a 15 participantes por variable en el modelo^36^. En este estudio, el tamaño de muestreo de 264 participantes atendió a la primera condición, pero impidió el análisis de la invariancia factorial del modelo. En el estudio original, la muestra fue de 310 estudiantes norteamericanos^3^; el estudio que validó el instrumento para la cultura coreana tuvo 284 participantes^14^ y el árabe, 389 participantes^30^. Todos emplearon el análisis factorial en sus análisis. 

En relación al análisis descriptivo del instrumento en cuestión, optamos por presentar la percepción de los participantes sobre el nivel de incivilidad y de su frecuencia por medio de la mediana, a pesar de que hubiésemos descrito la media para efecto de comparación con los estudios que validaron o emplearon ese instrumento en otras culturas^14^ ^),(^ ^30^. Este indicador (media) puede no representar la variación de la muestra con clareza, por tratarse de escala tipo Likert, por tanto, una escala cualitativa ordinal, según el nivel de mensuración^36^.

Comparando los niveles de incivilidad, para los comportamientos de estudiantes, en este estudio la media general fue de 3,0, en cuanto en los estudios coreano y árabe, estas fueron x = 3,11 y x = 3,45, respectivamente^14^ ^),(^ ^30^; y, para los comportamientos de profesores, los resultados de este estudio fueron x = 3,35 y los del estudio coreano x = 3,17^14^. Las medias de las frecuencias de ocurrencia de los comportamientos en los últimos 12 meses, referidas en este estudio para los comportamientos de los estudiantes y de profesores, fueron 2,10 y 1,82, respectivamente, en cuanto en el estudio árabe la media general para los ítems de los estudiantes fue 1,99^30^.

Además, el análisis descriptivo de los datos de este estudio, apunta aspectos que merecen ser considerados. Uno de ellos se refiere a la distribución de las respuestas. Fueron identificados los efectos techo-piso (*ceiling*/*floor effects*) para varios ítems del INE-R *Survey* versión brasileña. Esto indica que la distribución de los puntajes es asimétrica; o sea, retrata el porcentaje de los participantes que puntuaron los más bajos o elevados niveles de la medida^32^. En un estudio que utilizó el INE también se observó el efecto techo-piso habiendo más de 15% de frecuencia para la percepción de comportamientos de estudiantes y profesores en los dos polos de la escala utilizada^29^.

Se sabe que comportamientos disruptivos son más prevalentes que los inciviles de Alta Incivilidad^29^. Es más probable verificar comportamientos no verbales rudos y declaraciones de comentarios humillantes que comportamientos amenazadores o de actos violentos^3^.

La tendencia identificada de los participantes de atribuir un mayor nivel de incivilidad a un mismo comportamiento, cuando este es realizado por profesores, que cuando realizado por estudiantes, también ya había sido descrita en un estudio que analizó el efecto moderador de la jerarquía social en comportamientos inciviles en el trabajo^43^, mostrando que la incivilidad percibida fue mayor entre los actos incivilizados perpetrados por los gerentes en comparación con la incivilidad percibida si perpetrada por un compañero.

En relación a la validez y a la confiabilidad, la literatura apunta distintas alternativas para el análisis de las propiedades psicométricas, que dependen de la naturaleza de la variable, forma de medida, tamaño de la muestra y características de los datos obtenidos^36^. Además, siendo que ya existe un análisis exploratorio realizado por las autoras del instrumento^3^, se buscó confirmar esa propuesta, en la población de estudiantes brasileños, por medio de la validez de constructo, empleando la AFC, que es una técnica adecuada para probar (confirmar) si la estructura empírica observada en el conjunto de ítems presenta las mismas evidencias del constructo teórico de interés en la población analizada^44^. 

El estudio árabe con estudiantes de enfermería verificó, a partir de los resultados del análisis de tendencias y con validez aparente realizada por cuatro peritos, que algunos ítems retrataban intensidades distintas de incivilidad, en relación a la propuesta original norteamericana; y en la validez de constructo, por medio del Análisis Factorial Exploratorio, obtuvo cinco factores para la escala con comportamientos de estudiantes. El estudio no tuvo como objetivo la escala con ítems sobre comportamientos de docentes^30^. Este aspecto fue abordado en un estudio más reciente, utilizando los mismos procedimientos con 225 profesores, y que confirmó el modelo de 4 dominios para la escala que trata de comportamientos de los profesores^45^. 

Por otro lado, el estudio coreano, también con estudiantes, identificó que la escala con ítems de comportamientos de estudiantes presentaba, por medio del AFE, cuatro factores; y, la de profesores presentaba dos factores semejantes a la propuesta teórica original norteamericana^14^. 

El camino recorrido y los resultados descritos en este estudio indican que la propuesta teórica^3^ es válida para la población estudiada, en especial la existencia de dos dominios (alto nivel y bajo nivel de incivilidad) y también para la pertinencia de todos los ítems atribuidos a los comportamientos de profesores. 

Al analizar las cargas factoriales de los ítems, en cada dominio de comportamientos de profesores, se identificó un ajuste de todos los ítems con cargas elevadas de contribución para composición de los dominios. En contrapartida, al examinar los ítems que retratan los comportamientos de estudiantes, tres ítems no alcanzaron el valor 0,30, indicando su exclusión^46^. Sin embargo, para dos de ellos, ítems 5 y 15, en ambas AFCs, con y sin el ítem 1, los resultados estadísticos indican IC con límites superiores mayores que 0,30 y la opción fue por la manutención de ambos. Además, se refuerza que, cuando existe justificativa de orden teórica, un ítem con baja carga factorial puede ser mantenido o reformulado^46^.

Entre tanto, los bajos pesos factoriales, en el límite del punto de corte de 0,30, observados pueden estar asociados al tamaño de la muestra en cuestión; así, la manutención de los ítems 5 y 15, en la versión brasileña para estudiantes, necesita de más estudios con muestras diferentes. 

Los datos de las medidas de ajustes del modelo comprobado para los comportamientos de estudiantes (respectivamente, CFI = 1,000; 1,00; TLI = 1,000; NFI = 1,000; 1,000; RMSEA = 0,092; SRMR = 0,107) evidenciaron que los todos los índices indican un ajustamiento aceptable, excepto el SRMR. Para los profesores todos los índices fueron aceptables. 

Entre los comportamientos frecuentes relatados en la literatura^3^ ^)^ y concordantes con el enunciado teórico^25^ ^),(^ ^28^, se encuentran aquellos contenidos en los ítems que presentaron baja carga factorial en este estudio**.** Se destaca que un estudio norteamericano, realizado durante la pandemia, con 675 estudiantes de graduación y 35 profesores de enfermería^39^, identificó que los ítems 1 y 5 fueron los más frecuentes; igual resultado se observó en un estudio con 155 estudiantes y 40 profesores en Omán^47^.

Los comportamientos relacionados a los ítems 1, 5 y 15, pertenecientes al Dominio Baja Incivilidad, fueron percibidos como inciviles por los participantes de este estudio con las siguientes medias, considerando que los puntajes variaron de 1 a 4: ítem 1 ( x = 2,76); ítem 5 ( x = 3,12); e ítem 15 ( x = 2,21). Además, cuando se preguntó sobre la frecuencia de su ocurrencia, medida por los mismos parámetros, se presentaron las siguientes medias: ítem 1 ( x = 3,06); ítem 5 ( x = 3,40) e ítem 15 ( x = 2,27). 

Estos resultados mostraron que los alumnos observan esos comportamientos con relativa frecuencia, lo que apoya la manutención de los ítems en la escala, una vez que ellos son generalmente considerados incivilizados con base en los estudios anteriormente referidos.

En lo que se refiere a usar teléfono celular u otro dispositivo para finalidades no relacionadas con la actividad de la clase (ítem 5), fue considerado como falta de respeto para con el otro^28^; a pesar de que los estudiantes percibieron el comportamiento como incivil, permanecieron en contacto con otras personas o con *websites,* actitud similar a resultados de otros estudios realizados en diferentes culturas^30^ ^),(^ ^48^ ^)-(^ ^49^. Un estudio reciente sobre comportamientos de estudiantes, como el desarrollado en Canadá con profesores, también apunta que ese comportamiento es considerado disruptivo (para cerca de 80% de los participantes) y uno de los más frecuentes (para cerca de 60% de los participantes)^50^. En nuestro estudio, 54,5% de los estudiantes consideraron que esto ocurre frecuentemente y 33,3% que el mismo ocurre a veces. En lo referente al nivel de incivilidad, fue predominantemente considerado moderadamente incivil (46,2%) o altamente incivil (35,6%). Estos aspectos refuerzan la manutención de ese ítem en nuestro estudio. 

En relación al ítem 15 de exigir nuevas pruebas y ampliación de plazos, actitud común en las situaciones de desinterés en que no se está preparado para las actividades escolares^28^, el impacto de la dinámica de las actividades remotas empleadas en el período de realización de este estudio, pudo haber interferido en las expectativas de los alumnos frente a a situaciones de aprendizaje, ser posibles de cambiarlas a lo largo del tiempo y poder ser más flexible, en especial durante la pandemia. El ítem se refiere a exigir algo o no aceptar una respuesta negativa a un favor especial solicitado, en ese contexto se trata de comportamiento incivil. Este ítem, considerado por la mayoría de los estudiantes con bajo nivel de incivilidad (27,3% no incivil y 37,9% poco incivil), fue relatado como ocurriendo raramente (33,7%), nunca (26,5%), a veces (25,8%) o frecuentemente (14%). 

Entre los estudios que analizaron los efectos de la pandemia en la enseñanza de enfermería uno apuntó que las expectativas no realísticas y las dificultades de comunicación interfirieron negativamente en el aprendizaje en ese período, provocando desánimo y sentimiento de inadecuación^42^; otro mencionó el aprendizaje *online,* que durante ese período estresante, estaba relacionado con la baja satisfacción en aprender^51^. Además, el estudio ya referido apunt**ó** correlación entre aumento de estrés y comportamientos inciviles en ese período^39^.

Ese contexto también pudo haber interferido: en las respuestas emitidas, en las relaciones entre profesor y estudiante y en la motivación para asistir o participar de las actividades educacionales, aspectos considerados como distracciones o falta de respeto en la sala de clases^28^ y retratados en los resultados del presente estudio por medio de las respuestas al ítem 1 de la encuesta (ítem 1- expresar desinterés, aburrimiento o apatía sobre el contenido del curso o asunto). Entre los estudiantes, este ítem es observado predominantemente a veces (43,2%) o frecuentemente (33,7%), y es considerado prioritariamente como moderadamente incivil (47,3%) o altamente incivil (18,6%), reforzando la pertinencia de su inclusión en el instrumento. Estos aspectos apuntan la existencia de ese comportamiento y la percepción del nivel de incivilidad relativo al ítem para estudiantes, en la muestra. 

Los dos estudios que analizaron la validez de constructo para estudiantes, utilizaron el análisis factorial exploratorio, por considerar la falta de estudios de validez en la cultura árabe, así como la eventual influencia cultural en el instrumento^30^ o por no confirmar el modelo original propuesto de dos dominios, en la población coreana^14^. Ambos mantuvieron todos los ítems, tanto para los profesores como para estudiantes.

Otra medida aplicada al instrumento, con su estructura original de ítems, fue la verificación de la consistencia interna, la que se mostró altamente confiable para la muestra estudiada. Los valores obtenidos para los comportamientos de estudiantes (α= 0,937) y de profesores (α =0,973) son considerados como correlación casi perfecta. Los valores obtenidos en el estudio norteamericano fueron mayores que 0,96 para estudiantes y 0,98 para profesores^3^. En el estudio árabe^30^, el alfa fue 0,877 y en el coreano 0,940 para los ítems de estudiantes relacionados al nivel de incivilidad^14^. Se puede afirmar que el instrumento adaptado a la versión brasileña presentó consistencia interna para sus dominios de Baja y Alta Incivilidad para los comportamientos de estudiantes y de profesores, con valores altos también encontrados para alfa ordinal y omega de McDonald.

En relación a la reproductividad, se puede afirmar que el instrumento presentó reproductibilidad adecuada, verificada después 15 días, con 29 estudiantes. El número de participantes fue considerado aceptado y satisfactorio para el análisis, al evaluar que una muestra de tamaño n = 20 ya puede ser considerada suficiente para obtener un resultado de ICC adecuado^52^.

Al realizar el test-retest con 10 alumnos, los autores del estudio coreano obtuvieron un coeficiente de estabilidad de 0,73 para el nivel de incivilidad de estudiantes y 0,64 para el de profesores^14^. En este estudio, los ítems referentes a los estudiantes presentaron estabilidad buena (ICC = 0,612) y los relativos a los profesores, moderada (ICC = 0,598). Por otro lado, en relación a la estabilidad de los ítems que retratan la frecuencia de los comportamientos inciviles, observados en los últimos 12 meses, esta fue considerada moderada para los estudiantes (ICC = 0,474) y buena para los profesores (ICC = 0,614).

A pesar de que este estudio fue desarrollado apenas con estudiantes, la percepción de la presencia del fenómeno incivilidad, en el ambiente académico, merece reflexión; así, refuerza la necesidad de implantar procesos que estimulen las relaciones interpersonales y el diálogo sobre esa incivilidad; adoptar formas de enfrentamiento y, especialmente, como apuntado en un estudio^28^, asumir la responsabilidad de ambas partes. Cuando profesores y estudiantes buscan construir ambientes educacionales más respetables, el resultado es el crecimiento de la civilidad^28^. Experiencias educativas exitosas, con estimulo de la cultura de la civilidad, incluyendo el uso de aplicativos, han sido noticiadas^53^.

En lo que se refiere a la relevancia del estudio, cabe destacar que existe pertinencia del uso del INE-R-versión brasileña para la población estudiada, considerando: 1) la confiabilidad y estabilidad de la encuesta y 2) la adecuación integral a la propuesta teórica del instrumento original referente al número de dominios y a los ítems relacionados con los comportamientos de profesores en su totalidad y parcialmente en relación a los ítems de comportamiento de los estudiantes. La disponibilidad de una encuesta validada en el contexto estudiado, colabora con la ciencia de la Enfermería, al permitir identificar ese fenómeno o comparar los resultados en nivel internacional.

Por último, la percepción de los estudiantes, participantes de este estudio, sobre la existencia de los comportamientos abordados en el instrumento en análisis, refuerza la pertinencia de emplear el INE-R-versión brasileña para diagnosticar e intervenir en esa realidad, en los diferentes ambientes de la enseñanza. La correlación negativa entre comportamientos de incivilidad vividos por estudiantes y valores atribuidos a la profesión, identificada en un estudio iran**í**, se suma a esa preocupación^54^.

Se buscó realizar la presente investigación siguiendo las recomendaciones para estudios de esa naturaleza; sin embargo, fueron identificadas limitaciones como: la característica de la muestra ser de conveniencia; ser una única institución pública; y el número de participantes que inviabilizó el análisis de la invariancia de los datos en muestras aleatorias o grupos discriminantes. Ese número es explicado debido a que la recolección de dados, para este estudio, ocurrió a lo largo del tiempo de la pandemia. Hay que recordar que el criterio de inclusión en el estudio exigía la vivencia del estudiante por un año en el ambiente escolar; para algunos, ese plazo ocurrió, predominantemente, con actividades remotas, hecho que pudo haber potencializado la percepción del nivel y de la frecuencia de actos inciviles. 

Se sabe que el proceso de validación de una escala para culturas distintas es largo y exige pruebas en varios contextos^55^; delante de esto, se espera que esta estructura sea sometida al análisis psicométrico en nuevos estudios, con muestras de mayor dimensión y más generalizables para la realidad brasileña; y, entonces, poder decidir por la exclusión o no de los ítems 5 y 15, referidos en la versión para estudiantes podrá ser tomada. Este estudio fue desarrollado apenas con estudiantes; por tanto, es necesario validar el instrumento con profesores en caso de existir interés en emplearlo en esa población.

## Conclusión

El INE-R *Survey*-versión brasileña se encuentra validado para el contexto estudiado, con la confiabilidad descrita por el ICC, alfa de Cronbach, alfa Ordinal y omega de McDonald y la validez del constructo reiterada por la AFC, con el modelo ajustado para dos dominios (de alta y baja incivilidad) como previsto en su versión original. En relación a los ítems referentes a los comportamientos de profesores, hubo confirmación de todos ellos en relación al modelo teórico. Para los ítems relacionados a los comportamientos de estudiantes, los resultados apuntan para la exclusión, por ahora, del ítem 1 y la sugestión de realizar nuevas pruebas psicométricos de la versión para estudiantes, sin el referido ítem, en diferentes contextos.

El INE-R Survey - versión brasileña permite apoyar estudios y estrategias que necesitan medidas de percepción o de frecuencia de comportamientos inciviles en el ambiente de enseñanza superior de enfermería.
